# Evaluation of 6PPD-Quinone Lethal Toxicity and Sublethal Effects on Disease Resistance and Swimming Performance in Coastal Cutthroat Trout (*Oncorhynchus clarkii clarkii*)

**DOI:** 10.1021/acs.est.5c03697

**Published:** 2025-06-05

**Authors:** Prarthana Shankar, Ellie M. Dalsky, Joanne E. Salzer, Rachael F. Lane, Sophie Hammond, William N. Batts, Jacob L. Gregg, Justin B. Greer, Gael Kurath, Paul K. Hershberger, John D. Hansen

**Affiliations:** † Western Fisheries Research Center, 235175 U.S. Geological Survey, Seattle, Washington 98115, United States; ‡ Kansas Water Science Center, 2928 U.S. Geological Survey, Lawrence, Kansas 66049, United States; § Western Fisheries Research Center, 235175 U.S. Geological Survey, Marrowstone Marine Field Station, Nordland, Washington 98358, United States

**Keywords:** stormwater runoff, urban runoff mortality syndrome (URMS), tire road wear particles, 6PPD, 6PPDQ, salmon

## Abstract

6PPD-quinone (6PPDQ), derived from the tire-protectant 6PPD reacting with ozone, is an emerging contaminant of concern owing to its role in coho salmon (*Oncorhynchus kisutch*) deaths via urban runoff mortality syndrome (URMS). Given the impact of 6PPDQ on aquatic life in urban streams, we addressed the acute toxicity of 6PPDQ exposure on coastal cutthroat trout (CCT) (*Oncorhynchus clarkii clarkii*), a species sympatric with coho salmon in natal watersheds. Using static exposures coupled with analytical chemistry, we determined the 24-h LC_50_ values for alevin (297.2 ng/L), swim-up fry (39.6 ng/L), 5-month parr (103.3 ng/L), and 13-month juveniles (185.9 ng/L)—values similar to toxicity observed in coho salmon. Additionally, the 96-h LC_50_ (77.6 ng/L) was 2.4 times more lethal for juvenile CCT. We assessed potential effects of sublethal 6PPDQ exposure on disease resistance to infectious hematopoietic necrosis (IHN), an endemic viral disease of Pacific salmon, and to swimming performance. Sublethal 6PPDQ (53.6 ng/L) did not affect survival of parr exposed to IHN virus compared to virus alone. Conversely, 6PPDQ exposure as low as 72.2 ng/L significantly reduced 15- and 24-month juvenile swimming performance, and 120.5 ng/L 6PPDQ increased blood hematocrit. Overall, CCT are the second most sensitive species tested to date for 6PPDQ sensitivity which further emphasizes the need for identifying alternatives to 6PPD.

## Graphical abstract

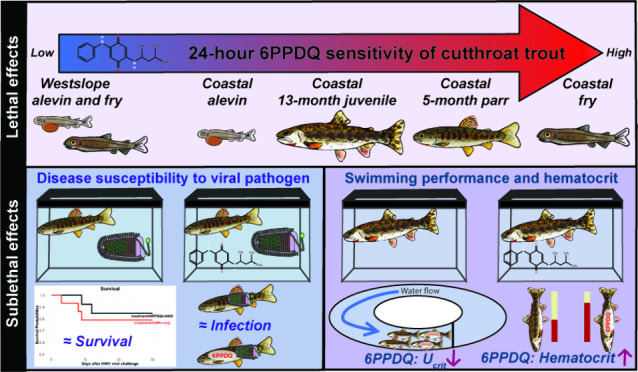

## 1 Introduction

Urbanization can alter aquatic and terrestrial ecosystems, causing notable harm to fish and wildlife through habitat fragmentation and pollution, with urban stormwater runoff introducing contaminants that can disrupt ecological processes. One such contaminant of concern is 6PPD-quinone [*N*-(1,3-dimethylbutyl)-*N*′-phenyl-*p*-phenylenediamine-quinone; 6PPDQ], an ozonated transformation product of 6PPD, a chemical additive used in rubber to prevent tire degradation. Recently, acute toxicity of 6PPDQ was documented in coho salmon (*Oncorhynchus kisutch*),^1^ in what is termed urban runoff mortality syndrome (URMS).^2^ Juvenile coho salmon have a reported 24-h median lethal concentration (LC_50_) of 95 ng/L,^3^ which is lower than environmental levels that can reach up to 2.43 μg/L in urban runoff.^4^

Since discovery of the acute toxicity of 6PPDQ, studies have explored effects in fish and invertebrates,^5^ and so far, only species in the *Salmonidae* family are known to be acutely sensitive (mortality) at environmentally relevant concentrations.^6–9^ Beyond identification of species-specific sensitivity differences,^10,11^ life stage may also influence sensitivity. Salmonid fry (life stage that emerges from gravel) appear to be more sensitive than older animals including postsmolt and postsmolt returns.^12,13^ Life stage dependence is ecologically relevant to salmonids, as different life stages could experience 6PPDQ exposure via urban stormwater runoff. In the Pacific Northwest (PNW) USA and British Columbia, many wild and hatchery-reared salmon and trout grow in urban-impacted streams, and they may return as adults to spawn where they may also be exposed to 6PPDQ. Further, depending on the species, animals spend varying lengths of time as juveniles in the streams before migrating out to sea.^14^ Thus, careful assessment of species susceptibility at different life stages is necessary.

One Pacific salmonid for which 6PPDQ sensitivity is not evaluated is coastal cutthroat trout (*Oncorhynchus clarkii clarkii*; CCT). One of four major North American cutthroat trout subspecies, CCT ranges from northern California to Alaska, and inhabits both coastal and inland waters.^15^ Depending on life history, juveniles spend 2–4 years in freshwater streams before migrating to sea, or freshwater lakes or streams.^16^ Of the other subspecies - westslope (*Oncorhynchus clarkii lewisii*; WCT), Lahontan (*Oncorhynchus clarkii henshawi*), and the Yellowstone cutthroat trout (*Oncorhynchus clarkii bouvieri*),^17^ only the WCT, a sister clade to the other subspecies,^18^ have been evaluated for 6PPDQ toxicity. Eight-month fish showed no signs of URMS or mortality at up to 10 μg/L 6PPDQ.^19^

Besides acute toxicity, few studies have addressed effects of 6PPDQ sublethal exposure in salmonids.^9,13,20,21^ This is likely driven in part by the low LC_50_ concentration in sensitive species, leading researchers to infer sublethal impacts from model organisms, such as zebrafish (*Danio rerio*) and *Caenorhabditis elegans* ^22,23^ that are not acutely sensitive to 6PPDQ at environmental concentrations. Thus, more research would be needed on sublethal effects in ecologically relevant species.

This study examines the lethal effects of acute 6PPDQ exposure on CCT across four life stages and investigates potential sublethal impacts on disease susceptibility to the infectious hematopoietic necrosis virus (IHNV) and swimming performance. IHNV, a single stranded RNA virus in the family *Rhabdoviridae*, is a major viral pathogen of Pacific salmon and trout, widespread throughout the PNW and associated with significant mortality.^24^ Swimming performance is crucial for survival, reproduction, and fish health.^25^ Overall, this study will enhance our understanding of 6PPDQ-related risks to salmonid fitness and survival that could have implications for the family *Salmonidae*.

## 2 Materials and Methods

### 2.1 Chemicals and Reagents

6PPD-quinone (purity 97.26%) and the isotopically labeled internal standard 6PPD-quinone-D5 were obtained from HPC Standards GmbH (Borsdorf, Germany) and the 6PPD-quinone analytical independent check standard from Cambridge Isotope Laboratories (Cambridge, MA, USA). Stock solutions of 6PPDQ were prepared using dimethyl sulfoxide (DMSO) (Thermo Fisher Scientific, Waltham, MA, USA) to achieve a final solvent concentration of <0.001% (v/v) during exposures. Buffered tricaine methanesulfonate (MS-222) (Syndel’s Syncaine, Ferndale, WA, USA) was used for anesthesia (100 mg/L) and euthanasia (250 mg/L).

### 2.2 Determination of Water Concentrations of 6PPDQ

One-milliliter water samples were collected in 1.85 mL amber borosilicate glass vials (Qorpak, Clinton, PA, USA) and stored at −20 °C until analysis at the U.S. Geological Survey (USGS) Kansas Water Science Center. The samples were analyzed for 6PPDQ using a direct-inject ultraperformance liquid chromatography mass spectrometry (UPLC-MS/MS) method.^26^

### 2.3 Fish Source and Culture

The primary CCT used in this study were obtained from the Washington Department of Fish and Wildlife (WDFW) Eells State Trout Hatchery near Shelton, WA. Fish from the same cohort were obtained as eyed eggs and as fry and transported to the USGS Western Fisheries Research Center (WFRC) in Seattle, WA. The eggs were utilized for 6PPDQ life-stage sensitivity determinations, and the fry were later evaluated for sublethal end points including disease susceptibility and swimming performance. Eggs were reared in egg stacks with spinning disc-filtered (2 μm final), UV-irradiated fresh water from Lake Washington (hereafter, wet laboratory water) maintained at 8 ± 1 °C. All fry and juveniles were reared in 2 or 2.5-ft diameter circular tanks (Reiff Manufacturing, Walla Walla, WA, USA) at 8 ± 1 °C. Fish were first started on BioVita Starter (Bio-Oregon, Warrenton, OR, USA) and then switched to Classic Fry feed (Skretting, Stavanger, Norway) until experiments.

CCT were also obtained from the WDFW Cowlitz Trout Hatchery near Toledo, WA (hereafter, CCT-Cowlitz). CCT-Cowlitz fish were approximately 15 g, and 7 months posthatch upon arrival, and animals were held at 10 ± 1 °C until juvenile 6PPDQ exposures for comparison with the CCT from the Eells State Trout Hatchery (Supplementary Method SM1).

WCT were obtained from the Sekokini Springs Hatchery near West Glacier, MT. Fish were obtained as eyed eggs, and similar to the CCT, were reared in egg stacks and then 2-ft tanks with wet laboratory water maintained at 8 ± 1 °C.

All experimental procedures were approved by the Animal Care and Use Committee at the USGS WFRC (Protocol #2008–72).

### 2.4 6PPDQ Life Stage Sensitivity Determination

Static exposures of 24 h to determine the 6PPDQ sensitivity of CCT at four life stages were conducted at the USGS WFRC in Seattle, WA, with wet laboratory water at 8 ± 1 °C (alevin, 2–5-week post swim-up fry, and 5-month parr exposures) or 10 ± 1 °C (13-month juvenile exposures). Individual tanks in all exposures had recirculating water around them for temperature maintenance. Except the alevin exposures, all aquaria were aerated with air stones. The parr, fry, and juveniles were last fed 48 h prior to chemical exposures. Solvent control tanks were set up with DMSO only, at the same DMSO concentration in the highest 6PPDQ exposure and never exceeded 0.001%. Survival in control tanks was always 100%.

Water samples were collected for analytical measurements immediately after addition of chemical (initial), and at the end of the 24-h exposure (final) (*n* = 16 for alevin, *n* = 13 for fry, *n* = 18 for parr, and *n* = 18 for juvenile). Tanks were also set up in the same way as the alevin, parr, and juvenile 24-h exposures without adding fish, to quantify reduction of chemical in the tanks over 24 h. Nominal 6PPDQ concentrations of 50 and 300 ng/L were used in the alevin and parr tanks, and 50 and 200 ng/L in the juvenile tanks for testing loss of 6PPDQ in absence of fish.

#### Alevin Exposures

The 24-h 6PPDQ alevin exposures began approximately 10 days posthatch, and experiments were completed within 2 weeks. CCT alevins (length and weight not measured) were exposed to 16 concentrations of 6PPDQ from 100 to 4000 ng/L (nominal). Static exposures without aeration were conducted in 2-L Pyrex beakers (Thermo Fisher Scientific, Waltham, MA, USA), each with 400 mL exposure solution and 20 alevins. The beakers were placed in 2-ft tanks which were covered for the 24-h duration of the exposure to ensure darkness. The total number of mortalities per beaker was determined at 24 h, and all remaining fish were euthanized.

#### Fry Exposures

Exposures to determine the 24-h LC_50_ were conducted within 3 weeks of each other using CCT that were 2–5 weeks post swim-up (fry) and weighed 0.15 ± 0.06 g standard deviation (SD) with 2.6 ± 0.3 cm SD fork length. Fry were exposed to 14 concentrations from 10–200 ng/L 6PPDQ (nominal). Static exposures occurred in 2.5-gallon glass aquaria (Aqueon, Franklin, WI, USA), with 8 L of exposure solution in each aquarium and six fish (0.11 g/L fish loading). Aquaria were covered with netting, aerated with a single air stone per aquarium, and fish were observed at least twice in the 24 h for URMS or mortality.

#### Age 5-Month Parr Exposures

Exposures to determine the 24-h LC_50_ were conducted within 3 weeks of each other using CCT that were 5-months posthatch (parr) and weighed 1.67 ± 0.39 g SD with 5.5 ± 0.5 cm SD fork length. Parr were exposed to 23 concentrations from 25–3000 ng/L 6PPDQ (nominal). Static exposures occurred in 9-L polycarbonate tanks (Aquatic Habitats), each with a total of 8 L of exposure solution and six fish (1.25 g/L fish loading). The 9-L tanks were covered with lids and fish were observed at least twice in the 24 h for URMS or mortality.

#### Age 13-Month Juvenile Exposures

Exposures to 13-month juveniles were performed to determine both 24-h and 96-h LC_50_ for CCT (12.0 ± 3.33 g SD; fork length of 9.8 ± 1.1 cm SD). All exposures were completed within 5 weeks of each other. Fish were exposed to 16 or 17 concentrations from 50–500 ng/L 6PPDQ (nominal), for 24-h and 96-h exposures, respectively. Exposures were conducted in 20-gallon glass aquaria (Aqueon, Franklin, WI, USA), with 50 L of exposure solution in each aquarium and six fish (1.44 g/L fish loading). Aquaria were covered with netting, and fish were observed for signs of URMS and mortality at least twice in the first 24 h.

For the 96-h LC_50_ determination, the Organization for Economic Co-operation and Development (OECD) Test Guideline No. 203–Fish Acute Toxicity Testing^27^ for semistatic exposures was followed. Fifty percent of the exposure solution was renewed once every 24 h (at 24, 48, and 72 h). On each renewal day, 25 L of the exposure solution was removed using a mini autosiphon (Fermtech Ltd., Ontario, Canada), and 25 L of 6PPDQ, at the same concentration as when the exposure started, was added back. Fish were fed once during the 96-h exposure approximately 23 h after exposure began. Excess food and waste were removed from the bottom of aquaria daily. Tanks were checked at least twice per day, mortalities were removed, and fish with signs of URMS were euthanized. For analytical confirmation, water samples from two tanks (100 and 200 ng/L nominal 6PPDQ) were collected at the beginning (initial), as well as pre- and postrenewal every 24 h, until 96 h post exposure start (final). One 96-h juvenile exposure (200 ng/L nominal 6PPDQ) was simulated without fish, to quantify reduction of 6PPDQ in the tank over 96 h.

Methods for CCT-Cowlitz and WCT 6PPDQ sensitivity determination, and the CCT pulsed exposures are described in Supplementary Method SM1.

#### Life Stage Sensitivity Data Analyses and Statistics

Data analyses were conducted using R scripts (version 4.3.0) written in RStudio (version 2024.04.2 Build 764).^28^ In addition to mortalities from 6PPDQ exposures, exposed fish showing clinical signs of URMS were euthanized and counted as mortalities. The percent mortality for each concentration was calculated at 24 h for the 24-h exposures, and at 24, 48, 72, and 96 h for the 96-h juvenile exposures. The drc R package^29^ was used for dose–response analysis at each life stage with LC_50_ estimations using a two-parameter log–logistic regression model (LL.2) fitted to percent survival against the analytically measured initial concentrations when available, or the nominal concentrations. The lower and upper limits were fixed at 0 and 1, respectively, and the model was specified with type = “binomial”. The survminer R package^30^ was used for Kaplan–Meier survival analysis for the 96-h daily mortality data.

### 2.5 Disease Susceptibility

Three experiments were conducted for optimization of methods to investigate the impact of 6PPDQ exposure on IHNV disease susceptibility in CCT (Supplementary Table S1). Methods are explained in detail in Supplementary Method SM2 and are briefly described here. All 6PPDQ and IHNV exposures to CCT were conducted at 10 ± 1 °C. Viral challenges in all three experiments included a 2-h static immersion challenge (*n* = 4 replicates, 19–21 fish/replicate) using a standard high dose of 2 × 10^5^ plaque-forming units (PFU)/mL of the virus followed by resumption of flow-through.

Experiment 1 was used to determine the susceptibility of CCT to the four most prevalent IHNV genetic subgroups in western North America (specific isolates in parentheses): UP (Blk94), UC (DW10), MD (Qts07), and L (FR0031),^24,31^ and to establish an IHNV disease model for CCT. Based on results from Experiment 1, Experiments 2 and 3 evaluated the effects of sublethal 24-h exposure to 6PPDQ on CCT susceptibility to IHN (MD strain, Qts07 isolate). Control groups exposed to only chemical or only virus were included, resulting in four treatment groups: Mock, 6PPDQ-only, IHNV-only, and 6PPDQ+IHNV. Seven-month fish were exposed to 50 ng/L (nominal) 6PPDQ (*n* = 4, 40 fish/65L; 1.2 g/L fish loading) followed by 24 h in freshwater, before the viral challenge (Experiment 2). The trial was revised slightly in Experiment 3, where 10-month CCT were exposed to 75 ng/L (nominal) 6PPDQ (*n* = 4, 40 fish/65L; 2.29 g/L fish loading) directly followed by the viral challenge. The sublethal 6PPDQ concentrations were selected based on the LC_50_ estimations from the life stage sensitivity determination exposures. In all three experiments, virulence was measured by observing signs of infection and mortality 30–36 days after the viral challenge, and infection was measured by quantifying the virus in head kidney tissue on days 3 and 7 (Experiment 1) or days 1 and 3 (Experiments 2 and 3) using RT-qPCR. Viral loads are expressed in copies of IHNV RNA/μg kidney RNA.

In Experiment 2, water samples (*n* = 4) were collected at the beginning (initial) and end of the 24-h exposure (final), as well as at 4 and 20 h for determination of measured 6PPDQ concentrations. In Experiment 3, water samples (*n* = 4) were collected at the start (initial) and at 24 h (final).

#### Disease Susceptibility Data Analyses and Statistics

Data analyses and visualization for the disease susceptibility experiments were conducted using RStudio. The survminer package^30^ was used for Kaplan–Meier survival analysis of mortality data gathered from three replicates of each treatment group in Experiments 1–3 with ggplot2 used for data visualization. The Cox proportional hazards test was conducted for identification of significant differences between treatment groups. For IHNV viral load data measured by RT-qPCR on two different days, mean log viral loads were compared across treatment groups and days of measurement (days 3 and 7 in Experiment 1 and days 1 and 3 in Experiments 2 and 3) using a two-way ANOVA test, followed by Tukey–Kramer test for post hoc analysis.

### 2.6 Swim Performance

All swimming performance trials and associated 6PPDQ exposures were conducted at the USGS WFRC, Marrowstone Marine Field Station in a custom-built swim flume (Supplementary Figure S1). Detailed experimental methods are provided in Supplementary Method SM3. Briefly, relative swim performance was assessed using a modified swimming respirometry trial protocol,^32^ whereby the water velocity in the flume was increased at prescribed intervals until all fish in the trial reached failure, defined as an inability to avoid impingement against the back of the swim chamber.

Pilot studies were performed to establish the water velocity ramping parameters for the experimental swim performance trials (Supplementary Table S2). Separate ramping parameters were developed for CCT at 15 months (14.9 ± 2.79 g SD and 115.82 ± 8.01 mm SD fork length; Experiment 4) and 24 months (29.4 ± 8.73 g SD and 147.17 ± 15.0 mm SD fork length, Experiment 5), with each protocol designed to fatigue all fish within 75 min. Experimental CCT were exposed for 22 h to 100 ng/L (nominal) 6PPDQ (1.83 g/L fish loading) [Experiment 4], or 150 ng/L (nominal) 6PPDQ (3.62 g/L fish loading) [Experiment 5]. Negative controls for each group were not exposed to 6PPDQ. Water samples were collected at the start (initial) (*n* = 6) and end (final) (*n* = 3) of the Experiment 4 exposures, and the start (initial) (*n* = 6) of the Experiment 5 exposures for determination of 6PPDQ concentration.

Fish were marked with color-coded elastomer tags to distinguish control and 6PPDQ-exposed groups. Each experiment included six swim trials, each with eight control and eight 6PPDQ-exposed fish. Water velocity was increased at the prescribed intervals until all fish achieved swim failure; time to failure was recorded for each fish, and fish were euthanized in buffered MS-222.

#### Hematocrit Measurements

Hematocrits were recorded from all fish in the first five trials of Experiment 5 using the HemataStat II (EKF Diagnostics, Boerne, TX, USA). A two-way ANOVA was performed to test the effect of the chemical on the interaction between hematocrit and *U*_crit_ values and the nonparametric Spearman’s rank correlation test was used to determine the correlation between the two variables.

#### Swim Performance Data Analyses and Statistics

Swimming performance was determined by estimating the average critical swimming speed (*U*_crit_) of the fish based on its time of fatigue and the corresponding water velocity in the flume. The *U*_crit_ formula has been described previously.^32,33^ Briefly, *U*_crit_ was calculated as the last full step velocity plus the temporal fraction of the step at fatigue. Fish with frayed fins or deformed bodies were excluded from the analysis. The *U*_crit_ values are presented in body lengths/second (BL/s), and were standardized by body length since total length of fish is the most important predictor of *U*_crit_.^25^

Replicate tanks of 6PPDQ treatments were tested for significant difference, and no significant differences were observed (one-way ANOVA test). Data were checked for normality (Shapiro test), and the Wilcoxon rank sum test for nonparametric data was used to compare significant differences between the controls and the 6PPDQ-treated fish in each Experiment.

## 3 Results and Discussion

### 3.1 Life Stage Comparison of 24-h Acute Toxicity in CCT

Acute toxicity of 6PPDQ was observed at all four CCT life stages. The mean 24-h LC_50_ concentration was 297.2 ng/L [95% confidence interval (CI) of 84.27–510.13 ng/L] in alevins, 39.6 ng/L [95% CI of 21.07–58.17 ng/L] in fry, 103.3 ng/L [95% CI of 38.86–167.71 ng/L] in 5-month parr, and 185.9 ng/L [95% CI of 108.18–263.67 ng/L] in 13-month juveniles (Figure 1A and Supplementary Figure S2A–D).

**Figure 1:**
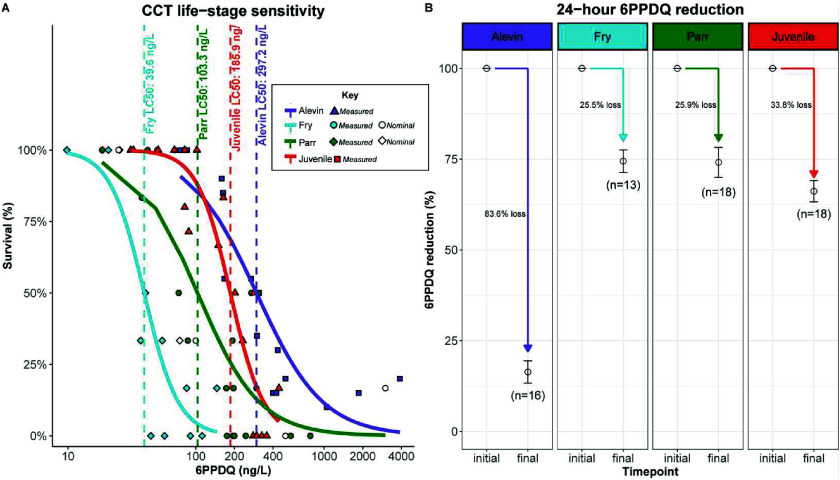
24-h 6PPDQ life stage sensitivity determination for CCT (*O. clarkii clarkii*). (A) 6PPDQ dose-response of the mortality data for CCT alevin, 2–5-week post swim-up fry, 5-month parr, and 13-month juveniles. Alevin (10–21 days post hatch) were exposed to 100–4000 ng/L 6PPDQ (nominal), fry (0.15 ± 0.06 g SD) were exposed to 10–200 ng/L 6PPDQ (nominal), 5-month parr (1.67 ± 0.39 g SD) were exposed to 25–3000 ng/L (nominal), and 13-month juveniles (12.0 ± 3.33 g SD) were exposed to 50–500 ng/L (nominal). When available, initial measured concentrations were used to generate the dose–response curves for each life stage to calculate their respective predicted 24-h LC_50_ concentrations, indicated by the vertical dashed lines. (B) Comparison of the percent decrease of 6PPDQ measured between the start and end of the alevin, fry, parr, and juvenile 24-h exposures. The number of individual exposure tanks used to calculate the percent reduction is indicated in each panel for each life stage.

Moribund alevins after 24 h, especially those exposed to 6PPDQ concentrations greater than 300 ng/L, demonstrated clinical signs of URMS including repeated oral gaping. These fish were also pale and slow to respond to touch. In addition, some alevin mortalities showed internal hemorrhaging in the brain, but clinical signs were not observed in all fish (example in Supplementary Figure S2E). Fry, parr, and juveniles showed the classic signs of URMS prior to mortality.^2^ Behavioral and external signs on moribund CCT included lethargic surface swimming and skin darkening for several hours prior to onset of URMS, which was always followed by mortality. Mortalities in parr began to occur within 4 h of exposure in treatments greater than 500 ng/L, and with most of the mortalities in treatments below 500 ng/L occurring within 14 h of exposure. Mortalities in the juvenile CCT generally occurred within 18 h of exposure. Overall, we did not observe a correlation between fish size and time of mortality, and mortalities occurred earlier in higher exposure concentrations, consistent with previously published work in other 6PPDQ-sensitive species.^7,9^

Our CCT results mirror the life-stage sensitivity differences identified in previous studies, including in coho salmon,^12^ lake trout (*Salvelinus namaycush*),^9^ and brook trout (*Salvelinus fontinalis*),^13^ where fry appear to be more sensitive than either age 1+ year juveniles or alevins. While previous studies did not compare more than two life stages within the same study, we found that CCT fry were over seven times more sensitive than alevin, and over 4.5 times more sensitive than the 13-month juveniles from the same fish cohort. The 2–5-week swim-up CCT fry were similarly sensitive to 3–4 week swim-up coho salmon fry in a previous study.^12^ Variation in individual fish sensitivity to 6PPDQ was also observed in CCT similar to other species;^12,13^ higher exposure concentrations in all life stages sometimes had fewer mortalities compared to lower exposure concentrations (Figure 1A and Supplementary Figure S2A–D).

### 3.2 Exposure Verification for 24-h Exposures

The 6PPDQ measured initial concentrations were on average 0.87×, 0.82×, 0.77×, and 0.86× the expected nominal concentrations, in the alevin, fry, 5-month parr, and 13-month juvenile exposures, respectively (Supplementary Table S3). Additionally, 6PPDQ waterborne concentrations declined throughout the exposure period in each experiment. The greatest decrease occurred in the alevin exposures with an average reduction of 83.6% (Figure 1B, possibly due to partial absorption by the lipid rich yolk sac). In comparison, 6PPDQ concentrations decreased by 25.5% in the fry exposures, 25.9% in the parr exposures and 33.8% in the juvenile exposures over 24 h. In a similar exposure design, when 40 parr were exposed in each of four 20-gallon tanks to 50 ng/L 6PPDQ (nominal) for Disease Susceptibility Experiment 2 below, measurements taken at 0, 4, 20, and 24 h revealed that majority of the 6PPDQ reduction occurred within the first 20 h of exposure (Supplementary Figure S3). Thus, the actual 24-h LC_50_ values are likely lower than the estimates presented here using the initial 6PPDQ measured concentrations. We also determined that the decrease in 6PPDQ was primarily due to fish uptake rather than their polycarbonate (parr) or glass (alevin, parr, and juvenile) exposure systems (Supplementary Figure S4). While the fate and distribution of 6PPDQ in salmonids remain unclear, one recent study found that 6PPDQ may be transformed into hydroxylated 6PPDQ which accumulates in the liver and gut of exposed rainbow trout (*Oncorhynchus mykiss*) fry.^34^ Similarly, a zebrafish study showed that embryos rapidly absorb 6PPDQ which undergoes extensive biotransformation, with over 95% of the absorbed compound transformed.^35^

The percent reduction of 6PPDQ measured in our study in the parr and juvenile exposures is lower than what has been reported previously in 24 h for juvenile rainbow trout (49.2–71.6%^36^) and juvenile white-spotted char (*Salvelinus leucomaenis*) (47–97%^8^), two species considered to be acutely sensitive to 6PPDQ. An average loss of 14% (between 1.7% and 21%) was reported by Brinkmann et al.^7^ in their 24-h 6PPDQ exposures with larger salmonids (>50.0 g), suggesting that while fish uptake is possible over the 24-h exposure, the decrease of 6PPDQ can be influenced by experimental methods including fish loading, size and species of fish, and sensitivity of the test species.

### 3.3 Comparison of CCT with Other Salmonids

The 24-h LC_50_ values indicate that CCT are currently the second most sensitive species to 6PPDQ exposure, and that 5-month CCT parr are as sensitive as juvenile coho salmon, while the fry of both species are similarly sensitive (Table 1). We corroborated our results by showing that 8–11-month juvenile CCT from a different hatchery (CCT-Cowlitz) exhibited similar sensitivity to 6PPDQ in a head-to-head comparison with 20–23-month CCT from Eells State. CCT-Cowlitz had an LC_50_ of 133.6 ng/L [95% CI of 61.03–206.14 ng/L] compared to Eells State CCT’s 24-h LC_50_ of 211.1 ng/L [95% CI of 85.08–337.14 ng/L] (Supplementary Figure S5). Cowlitz-CCT are likely equally or more sensitive than the Eells State CCT, confirming high 6PPDQ sensitivity of CCT across two different populations.

**Table 1:**
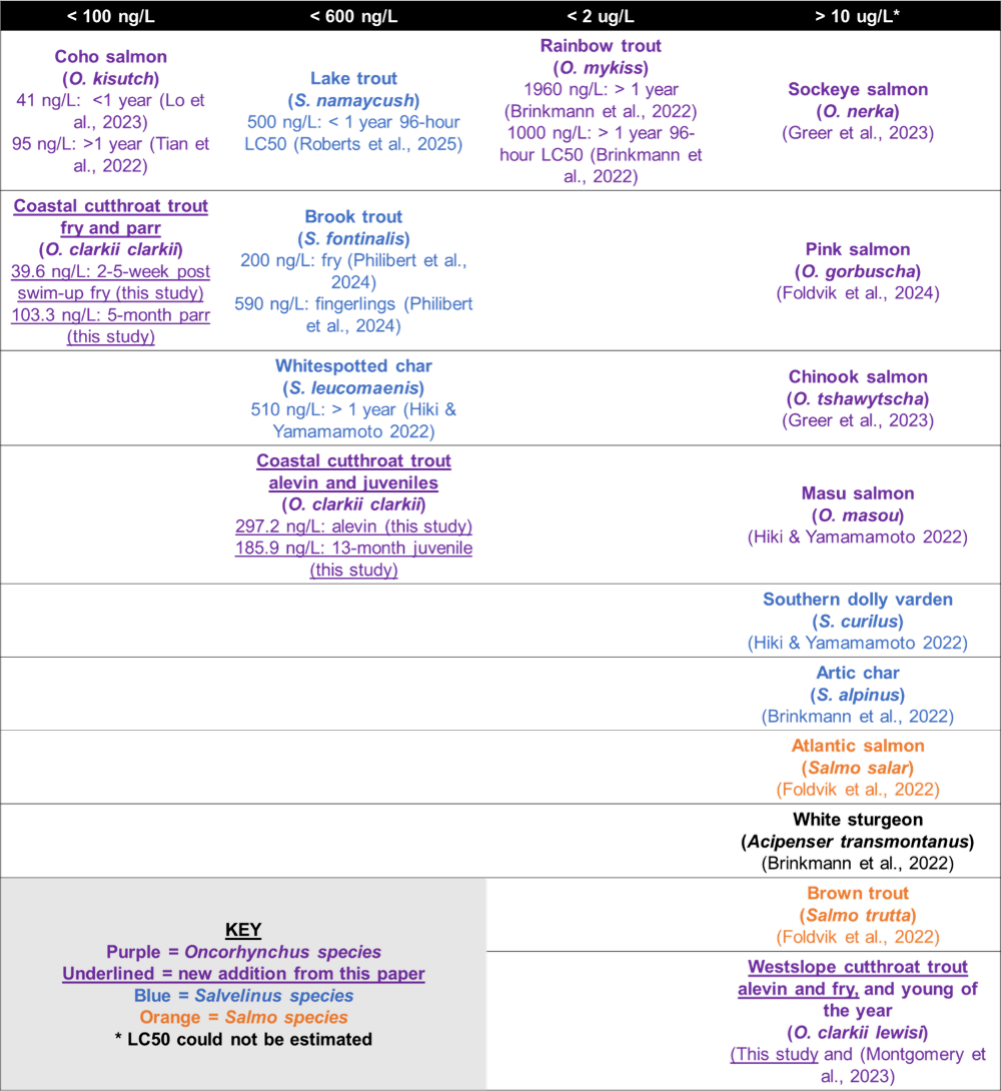
Salmonid Fish Species Organized by Their Determined 6PPDQ 24-h LC_50_ Values from the Literature^a^

a The newly identified coastal cutthroat trout (*O. clarkii clarkii*) 24-h LC_50_ values are included for comparison with other species.

In much of the PNW USA and British Columbia, CCT often reside in urban streams inhabited by other salmonids such as coho salmon and steelhead (*O. mykiss*).^37^ While URMS in CCT has not been reported in the wild, recent studies have described declines in subpopulations of this species in native streams where they have to coexist with stressors such as predation, habitat degradation, and environmental contaminants.^38,39^ Importantly, sexual maturity in CCT is reached between 2 and 3 years posthatch, with spawning typically occurring from December to May, peaking in April in the PNW. Once mature, individuals are iteroparous and practice repeat spawning for multiple years,^16^ which could contribute to a higher resilience to environmental chemical toxicity, including exposure to 6PPDQ.

Different subspecies of cutthroat trout demonstrate dramatically different sensitivities to 6PPDQ, with CCT being highly sensitive, and WCT being largely insensitive. WCT alevin and 3–4 week-swim-up fry exposed to 10 μg/L and 3 μg/L 6PPDQ, respectively, did not show signs of URMS or mortality.^40^ These results align with previous findings,^19^ and are noteworthy, as our WCT were sourced from Sekokini Springs Hatchery near West Glacier, MT, USA, and the study by Montgomery et al.,^19^ used fish from the Allison Creek Trout Hatchery in Coleman, Alberta, Canada. Despite the different origins, results from both populations were consistent. While closely related species, such as, Chinook (*Oncorhynchus tshawytscha*) and coho salmon, have been previously shown to have completely different sensitivities to 6PPDQ (Table 1), our study addressing early life stages would be the first instance where two subspecies (CCT and WCT) have been identified to have greatly different acute sensitivities to 6PPDQ.

### 3.4 96-h Acute Toxicity in 13-Month Juvenile CCT

The 13-month juvenile CCT 96-h LC_50_ in this study was 2.4-fold lower than the 24-h LC_50_ (185.9 ng/L; Figure 1B). The estimated 96-h LC_50_ was 77.6 ng/L [95% CI of 27.04–128.10 ng/L; Figure 2A]. After the initial dosing of the tanks, our 96-h semistatic exposure method resulted in between 0.4–0.8 x of the initial measured 6PPDQ concentration after the last (72 h) 50% renewal (Supplementary Figure S6). To date, only a few studies in rainbow trout have reported 96-h LC_50_ values for 6PPDQ; one study estimated 96-h LC_50_s between 1660–4310 ng/L depending on the enantiomer of 6PPDQ,^41^ which is corroborated by the 96-h LC_50_ of 1000 ng/L determined in another study.^7^ Brinkmann et al. identified a nearly 2-fold difference between the 24- and 96-h LC_50_s for rainbow trout. Mortalities in our study occurred throughout the duration of the 96-h exposure (Figure 2B) and was different from the 60-h mortality timeline reported for rainbow trout^7^ and the juvenile lake trout timeline, where mortalities occur only in the first 24 h.^9^ While differences in exposure designs and life stages may have an impact, there may be species-specific timelines of mortality after 6PPDQ exposure resulting from slight differences in chemical uptake and resulting toxicity mechanisms between sensitive species.

**Figure 2:**
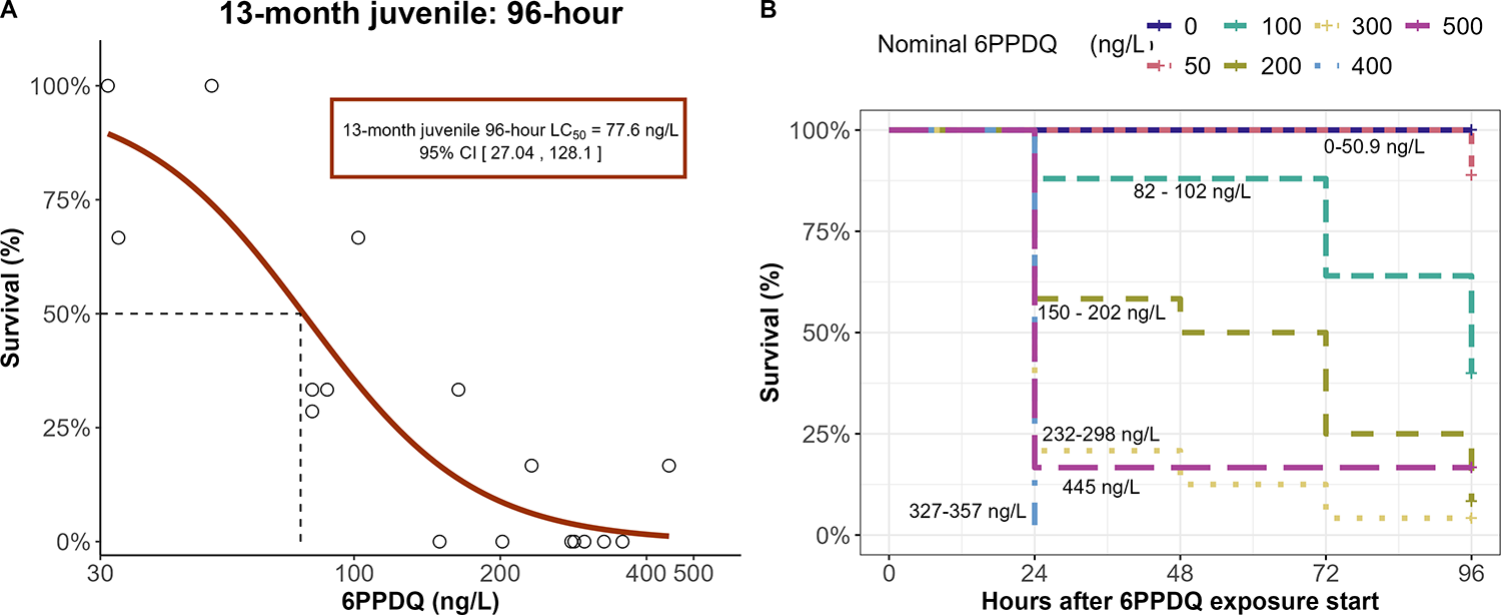
96-h semistatic 6PPDQ exposure to 13-month juvenile CCT (*O. clarkii clarkii*) (A) Dose response curve with estimated 96-h median lethal (LC_50_) concentration for 13-month juvenile CCT generated from exposures conducted between 31.1 and 445 ng/L. (B) Average survival measured every 24 h of the 96-h exposure. Each curve represents the average survival of the measured concentration treatments indicated near the respective curves.

While the differences between the 24-h and 96-h LC_50_ values for the CCT is intriguing, a more environmentally relevant scenario would be to mimic intermittent rainfall events common in the PNW. No difference in mortality was observed compared to the semistatic 96-h exposure at the same approximate concentrations (Supplementary Figure S7). The results suggest that CCT exposed to 6PPDQ at sublethal levels may have a limited ability to recover from toxic effects before additional exposures.

### 3.5 6PPDQ Impacts to IHN Susceptibility in CCT

A laboratory-based IHN challenge model for CCT was successfully developed (Experiment 1) to test the hypothesis that sublethal 6PPDQ exposure would render fish more susceptible to virus-mediated disease (Experiments 2 and 3) (Supplementary Table S1). Of the four IHNV genetic subgroups (UP, UC, MD, and L) tested, MD had the highest virulence, infectivity, and persistence in 5-month parr CCT (Supplementary Figure S8 and Table S4). We also noted that the MD-infected fish demonstrated the clinical signs of IHNV infection^42^ including skin darkening and hemorrhaging (Supplementary Figure S10). Results indicated that CCT were only moderately susceptible to IHN (28% mortality; *p* = 0.022), consistent with early, preliminary reports that cutthroat trout are less susceptible to IHNV than rainbow trout.^43,44^ Thus, we selected the MD strain for our development of a viral challenge model to assess potential sublethal effects of 6PPDQ on disease susceptibility.

Prior exposure to sublethal levels of 6PPDQ did not significantly increase susceptibility to the viral disease (Figure 3A and Supplementary Figure S9A). Contrary to expectation, the 6PPDQ+IHNV group had slightly higher survival than the IHNV-only group in both experiments. We did not observe any differences in severity or frequency of clinical signs between the IHNV-only and 6PPDQ+IHNV treatment groups in either experiment (data not shown). In Experiment 3, virus titers were similar in 7/12 of the IHNV-only mortalities and 7/9 of the 6PPDQ+IHNV mortalities, with average virus titers of 8.10 × 10^5^ PFU/g and 6.5 × 10^5^ PFU/g of fish, respectively. On day 1 of Experiment 3, 60% of the IHNV-only fish were infected with virus, compared to 30% of the 6PPDQ+IHNV fish (Figure 3B). On day 3, almost all fish (100 or 90%, respectively) were infected with the virus. Viral loads were higher in the IHNV-only group on both days, with over a 10-fold difference between the two treatment groups on day 3 (not statistically significant). In Experiment 2, differences in infection level between groups on both days matched results of Experiment 3 (Supplementary Figure S9B).

**Figure 3:**
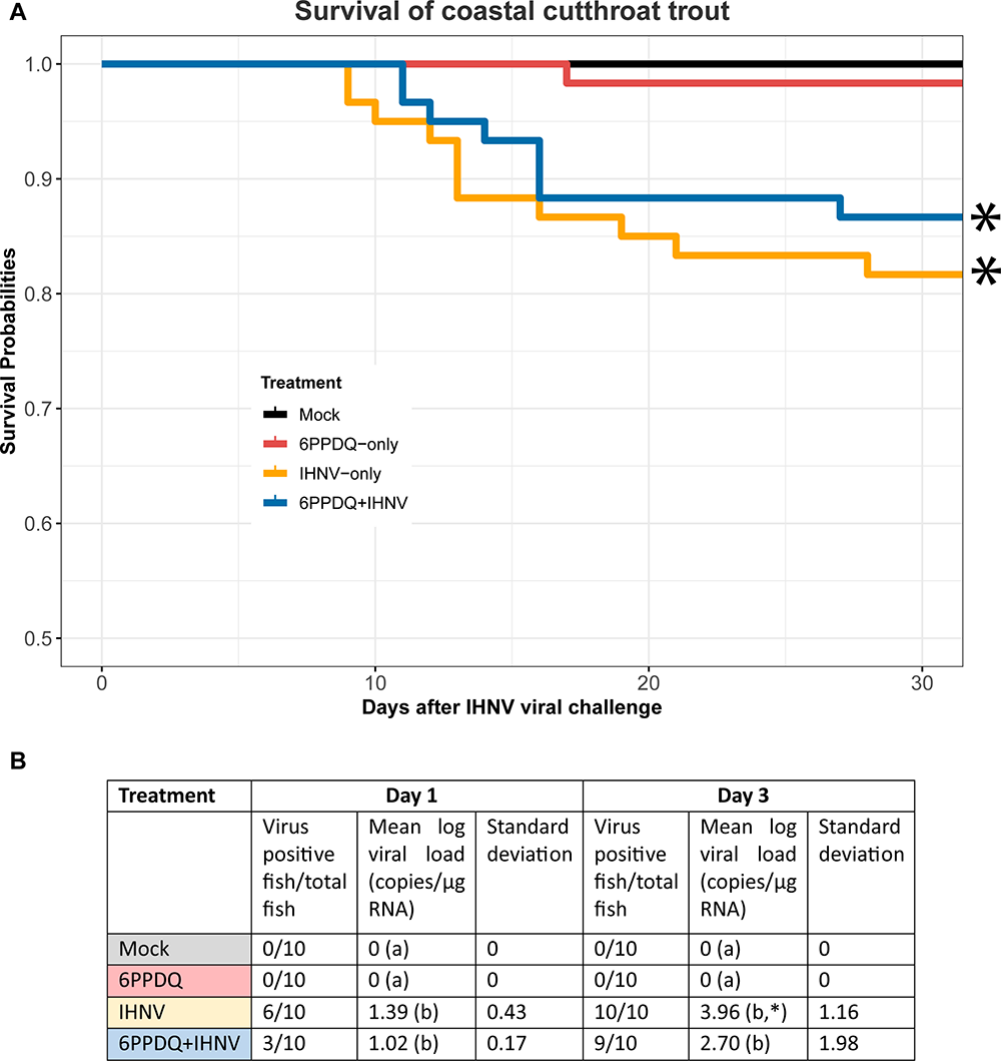
Effects of 6PPDQ exposure on CCT (*O. clarkii clarkii*) susceptibility to IHNV. (A) Daily cumulative percent survival of 10-month CCT following exposure to 6PPD-only, IHNV-only, 6PPDQ+IHNV, and a Mock treatment group (Experiment 3). The 6PPDQ initial concentration was 53.6 ± 4.9 ng/L. The virus exposure following the 24-h chemical exposure was a 2 h static immersion challenge to a standard high dose of 2 × 10^5^ plaque-forming units (PFU)/mL of the MD strain, Qts07. The Mock group (control) was exposed to an equal DMSO % as the 6PPDQ treatment, followed by virus-free media during the viral challenge. Survival curves were constructed by pooling data from triplicate groups (*n* = 19–21) of each treatment. Asterisk indicates that the two IHNV groups had significantly lower survival compared to fish in the Mock treatment (*p* < 0.05, Cox proportional hazard analysis). No differences were observed between the two IHNV groups (IHNV-only and 6PPDQ+IHNV), or between 6PPDQ-only and the Mock groups. (B) Summary of CCT infection frequencies and log_10_ viral loads in 10 fish/treatment group (Mock, 6PPDQ-only, IHNV-only, 6PPDQ+IHNV) on days 1 and 3 post viral challenge. Different letters in parentheses indicate treatment groups with mean log viral loads that are significantly different from each other on each day, and asterisk indicates statistical significance between days 1 and 3 of the same treatment group (*p* < 0.05; two-way ANOVA; Tukey post hoc test).

Preceding the viral challenge, we measured an average initial 6PPDQ concentration of 37.3 ng/L in Experiment 2, and 53.6 ng/L in Experiment 3 (Supplementary Table S5 and Figure S3), concentrations well within the range of what has been measured in stormwater from the PNW.^3,26^ Based on the life stage sensitivity experiment for the 5-month parr life stage (Figure 1A and Supplementary Figure S2C), the exposure concentrations in Experiments 2 and 3 were approximately the LC_14_ and LC_23_ values, respectively (Supplementary Table S6). One fish with signs of URMS was identified in Experiment 2 and none in Experiment 3. It is possible that the older Experiment 3 fish were slightly less sensitive to 6PPDQ; based on the 13-month juvenile sensitivity, fish were exposed to < LC_2_ concentration (53.6 ng/L) (Figure 1A and Supplementary Figure S2D and Table S6).

Overall, results indicate that sublethal 6PPDQ exposure did not significantly alter IHNV disease susceptibility in CCT. Both the slightly lower mortality and the lower infection (lower number of infected fish and lower viral loads) in the 6PPDQ+IHNV treatment group compared to IHNV-only suggest that 6PPDQ exposure may have a slightly protective, corollary effect against the virus. It is possible that an immune system component may have been primed by 6PPDQ exposure, thereby reducing the impact of the IHNV challenge. Our previous study using embryonic coho salmon showed a dose–response increase of proinflammatory genes after exposures to 6PPDQ.^20^ Similarly, a rodent study investigating impacts of dietary 6PPD and 6PPDQ exposure on liver inflammation observed significant increases in inflammatory-related genes including tumor necrosis factor-α from 6PPDQ exposure^45^ highlighting the potential of 6PPDQ exposure to influence the immune system in multiple organisms. While the immune system may not be a primary target of acute 6PPDQ exposure in salmonids, individual fish variability of 6PPDQ mediated effects may have masked our ability to detect significant impacts on IHN susceptibility. Further, it is possible that majority of the 6PPDQ was biotransformed in the fish before the viral exposure occurred;^34,35^ future studies investigating 6PPDQ-pathogen coexposures may provide further insights.

### 3.6 6PPDQ Impacts to the CCT Swimming Performance and Hematocrit

After addressing the potential sublethal effects of 6PPDQ on disease resistance, we investigated the potential for 6PPDQ to interfere with swimming performance in two experiments (Supplementary Table S1). Sublethal, 22-h exposure to 6PPDQ significantly reduced CCT swimming performance. CCT exposed to 72.2 ng/L and 120.5 ng/L 6PPDQ had significantly lower (*p* = 0.007 and 0.035) average *U*_crit_ values compared to control fish in each experiment (Figure 4A). The *U*_crit_ values of 6PPDQ-exposed fish were 4.03 ± 0.28 SD and 3.12 ± 0.62 SD BL/s, in Experiment 4 and 5, respectively, and control *U*_crit_ values were 4.16 ± 0.26 SD and 3.40 ± 0.32 SD BL/s; the lower control *U*_crit_ values in the latter case likely explained by the older 24-month CCT in Experiment 5 (Experiment 4 fish were 15-month-old). When examining individual fish, the difference between control and 6PPDQ-exposed fish was most pronounced in trial 1 of each experiment (Supplementary Figure S11).

**Figure 4:**
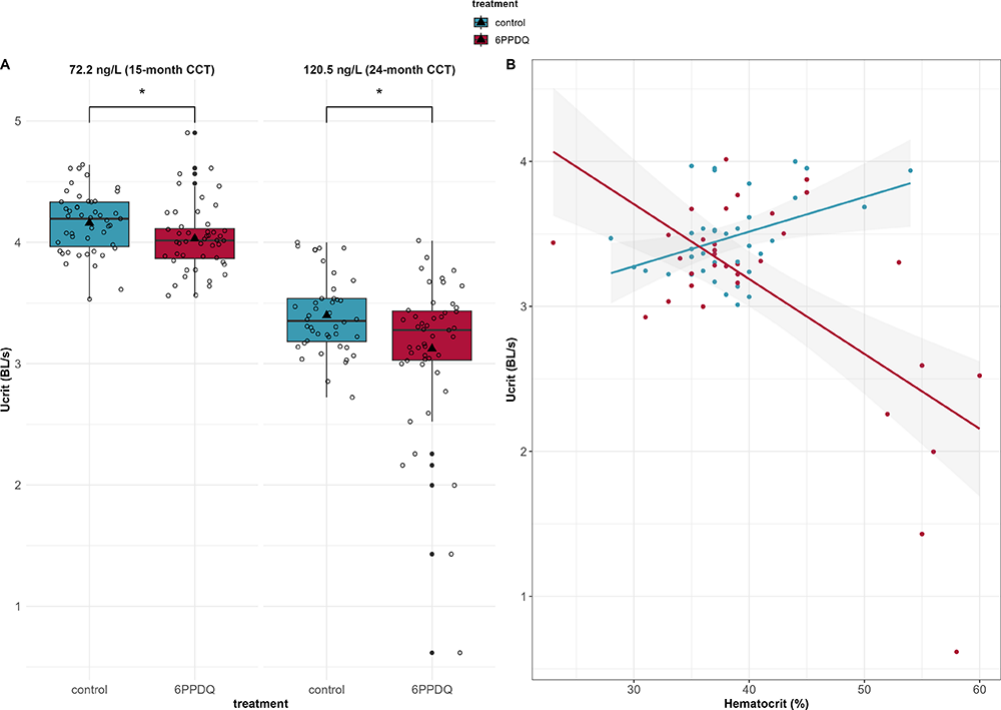
Swimming performance and hematocrit of juvenile CCT *(O. clarkii clarkii*) exposed to 6PPDQ (A) Critical swimming speed (*U*_crit_) of 15-month CCT exposed to 72.2 ng/L or 24-month CCT exposed to 120.5 ng/L 6PPDQ for 22 h compared to corresponding control fish exposed to the equivalent % DMSO concentrations. Swimming speeds are presented as body lengths per second (BL/s) (box and whisker plot). Center line of the plot = median; triangle = mean; box = interquartile range; whiskers = 1.5× interquartile range; dark points = values outside the 1.5× interquartile range, light points = *U*_crit_ values for individual fish. The nonparametric Wilcoxon rank sum test was used to compare control vs 6PPDQ-exposed fish at each concentration (*p < 0.05). (B) Scatter plot depicting the relationship between hematocrit (%) and *U*_crit_ (BL/s) in control and 150 ng/L 6PPDQ-treated 24-month juvenile CCT. Trend lines represent the linear regression fit for each treatment group, with shaded areas indicating the 95% confidence intervals.

Measured initial 6PPDQ concentrations in Experiment 4 ranged from 50.4 to 81.3 ng/L across the six trials (Supplementary Table S7), an average of 72.2 ng/L or the LC_4_ (Figure 1A and Supplementary Figure S2D and Table S6). In agreement with the LC_50_ curve for juvenile CCT (Figure 1A), none of the exposed fish exhibited signs of URMS. The initial 6PPDQ concentrations in Experiment 5 ranged from 93.1 to 135.0 ng/L, an average of 120.5 ng/L or the LC_19_ (Figure 1A and Supplementary Figure S2D and Table S6). One fish showed classic signs of URMS (loss of equilibrium and gaping; trial 1) and two fish (trials 2 and 4) were beginning to swim at the surface. All fish were included in the swimming performance test and subsequent analyses.

The current work, along with at least one previous study in juvenile lake trout,^21^ collectively suggest that 6PPDQ exposure for less than 24 h at environmental levels can significantly reduce salmonid swimming performance. Additionally, 6PPDQ causes cardiovascular dysfunction in zebrafish larvae,^46^ and disrupts vascular permeability molecular pathways in coho salmon embryos,^20^ all of which suggest 6PPDQ-induced cardiovascular stress that could affect swimming performance.

We also found a significant interaction (*p* < 0.0001) between hematocrit and treatment, indicating that the effect of hematocrit on *U*_crit_differed between the control and 6PPDQ-exposed CCT (Figure 4B). Control fish showed a slight positive trend (*p* = 0.08) in the correlation between *U*_crit_ and hematocrit, while 6PPDQ-exposed fish showed a slight negative trend (*p* = 0.18). Importantly, the 6PPDQ-related negative trend was only evident in individuals with hematocrit greater than 50% (where over half of blood volume is composed of red blood cells, RBCs), and includes the three fish with signs of URMS. Our single concentration 6PPDQ experiment suggests that while hematocrit is influenced by 6PPDQ, hematocrit may not be a reliable biomarker for sublethal 6PPDQ exposure.

Two possible explanations for increase in hematocrit after 6PPDQ exposure is the release of RBCs from the spleen, a mechanism that occurs during high-stress situations to enhance oxygen delivery,^47^ or through fluid leakage from vessels resulting from vascular permeability. Our results mirror significant concentration-dependent increases in hematocrit that have been observed in brook trout and rainbow trout with 6PPDQ,^7,13^ and in coho salmon exposed to roadway runoff.^48^ It is suggested that a significant rise in hematocrit, signaling severe plasma loss, can be a sufficient pathological factor contributing to acute mortality in coho salmon.^49^

Taken together, our results demonstrate that 6PPDQ is acutely toxic to multiple life stages of CCT at environmentally relevant concentrations; especially for young CCT (swim-up fry and 5-month parr) which are equally as sensitive as coho salmon. Additionally, 6PPDQ negatively affects swimming performance, specifically reducing critical swimming velocity. In contrast, sublethal 6PPDQ exposure does not significantly increase susceptibility to a viral pathogen. Overall, this study provides further evidence for the toxicity of 6PPDQ to aquatic animal species. While the long-term ecological impacts of 6PPDQ exposure in CCT remain unknown, these results along with others highlight the potential for population-level effects in Pacific salmonids, particularly across multiple vulnerable life stages in urban-impacted streams. Identifying safer alternatives to 6PPD in tire manufacturing could help protect aquatic ecosystems.

## Supplementary Material





## Data Availability

All data presented in this manuscript are available in the U.S. Geological Survey data release:^40^ 10.5066/P16SMKIJ.

## Notes

Additional experimental methods and images, and an overview of the sublethal experiments, measured 6PPDQ concentrations in all experiments, estimated LC values for the four CCT life stages, and the individual 6PPDQ dose response curves for each CCT life stage, figures for CCT-Cowlitz, pulsed exposures, and additional figures for 6PPDQ percent reduction in the water (PDF)
Additional values (XLSX)

## References

[1] Tian Z., Zhao H., Peter K. T., Gonzalez M., Wetzel J., Wu C., Hu X., Prat J., Mudrock E., Hettinger R., Cortina A. E., Biswas R. G., Kock F. V. C., Soong R., Jenne A., Du B., Hou F., He H., Lundeen R., Gilbreath A., Sutton R., Scholz N. L., Davis J. W., Dodd M. C., Simpson A., McIntyre J. K., Kolodziej E. P. (2021). A ubiquitous tire rubber-derived chemical induces acute
mortality in coho salmon. Science.

[2] Scholz N. L., Myers M. S., McCarthy S. G., Labenia J. S., McIntyre J. K., Ylitalo G. M., Rhodes L. D., Laetz C. A., Stehr C. M., French B. L., McMillan B., Wilson D., Reed L., Lynch K. D., Damm S., Davis J. W., Collier T. K. (2011). Recurrent
die-offs of adult coho salmon returning to spawn in Puget Sound lowland
urban streams. PloS ONE.

[3] Tian Z., Gonzalez M., Rideout C. A., Zhao H. N., Hu X., Wetzel J., Mudrock E., James C. A., McIntyre J. K., Kolodziej E. P. (2022). 6PPD-Quinone:
Revised Toxicity Assessment and Quantification
with a Commercial Standard. Environmental Science
& Technology Letters.

[4] Cao G., Wang W., Zhang J., Wu P., Zhao X., Yang Z., Hu D., Cai Z. (2022). New Evidence
of Rubber-Derived
Quinones in Water, Air, and Soil. Environ. Sci.
Technol..

[5] Prosser R. S., Salole J., Hang S. (2023). Toxicity of 6PPD-quinone to four
freshwater invertebrate species. Environ. Pollut..

[6] Chen X., He T., Yang X., Gan Y., Qing X., Wang J., Huang Y. (2023). Analysis, environmental
occurrence, fate and potential toxicity of
tire wear compounds 6PPD and 6PPD-quinone. Journal
of Hazardous Materials.

[7] Brinkmann M., Montgomery D., Selinger S., Miller J. G. P., Stock E., Alcaraz A. J., Challis J. K., Weber L., Janz D., Hecker M., Wiseman S. (2022). Acute toxicity of the
tire rubber-derived
chemical 6PPD-quinone to four fishes of commercial, cultural, and
ecological importance. Environ. Sci. Technol.
Lett..

[8] Hiki K., Yamamoto H. (2022). The Tire-Derived Chemical 6PPD-quinone
Is Lethally
Toxic to the White-Spotted Char Salvelinus leucomaenis pluvius but
Not to Two Other Salmonid Species. Environmental
Science & Technology Letters.

[9] Roberts C., Lin J., Kohlman E., Jain N., Amekor M., Alcaraz A. J., Hogan N., Hecker M., Brinkmann M. (2025). Acute and
Subchronic Toxicity of 6PPD-Quinone to Early Life Stage Lake Trout
(Salvelinus namaycush). Environ. Sci. Technol..

[10] Foldvik A., Kryuchkov F., Ulvan E. M., Sandodden R., Kvingedal E. (2024). Acute Toxicity Testing of Pink Salmon (Oncorhynchus
gorbuscha) with the Tire Rubber–Derived Chemical 6PPD-Quinone. Environ. Toxicol. Chem..

[11] Greer J. B., Dalsky E. M., Lane R. F., Hansen J. D. (2023). Establishing an
in vitro Model to Assess the Toxicity of 6PPD-Quinone and Other Tire
Wear Transformation Products. Environmental
Science & Technology Letters.

[12] Lo B. P., Marlatt V. L., Liao X., Reger S., Gallilee C., Ross A. R. S., Brown T. M. (2023). Acute Toxicity
of 6PPD-Quinone to
Early Life Stage Juvenile Chinook (Oncorhynchus tshawytscha) and Coho
(Oncorhynchus kisutch) Salmon. Environ. Toxicol.
Chem..

[13] Philibert D., Stanton R. S., Tang C., Stock N. L., Benfey T., Pirrung M., de Jourdan B. (2024). The lethal and sublethal impacts
of two tire rubber-derived chemicals on Brook trout (Salvelinus fontinalis)
fry and fingerlings. Chemosphere.

[14] Wydoski, R. S. ; Whitney, R. R. Inland Fishes of Washington; University of Washington Press, 2003.

[15] Behnke, R. J. Native Trout of Western North America; American Fisheries Society Monograph (USA); American Fisheries Society, 1992; No. 6.

[16] Trotter P. C. (1989). Coastal
cutthroat trout: a life history compendium. Transactions of the American Fisheries Society.

[17] Page, L. M. Common and Scientific Names of Fishes from the United States, Canada, and Mexico, 7th ed.; American Fisheries Society, 2013.

[18] Kokkonen A. L., Searle P. C., Shiozawa D. K., Evans R. P. (2024). Using de novo transcriptomes
to decipher the relationships in cutthroat trout subspecies (Oncorhynchus
clarkii). Evol Appl..

[19] Montgomery D., Ji X. W., Cantin J., Philibert D., Foster G., Selinger S., Jain N., Miller J., Mcintyre J., de Jourdan B., Wiseman S., Hecker M., Brinkmann M. (2023). Interspecies
Differences in 6PPD-Quinone Toxicity Across
Seven Fish Species: Metabolite Identification and Semiquantification. Environ. Sci. Technol..

[20] Greer J. B., Dalsky E. M., Lane R. F., Hansen J. D. (2023). Tire-Derived Transformation
Product 6PPD-Quinone Induces Mortality and Transcriptionally Disrupts
Vascular Permeability Pathways in Developing Coho Salmon. Environ. Sci. Technol..

[21] Selinger S., Hunnie B., Roberts C., Amekor M., Hogan N., Wiseman S., Hecker M., Weber L., Janz D., Brinkmann M. (2025). Sublethal
6PPD-quinone exposure impairs swimming performance
and aerobic metabolism in juvenile lake trout (Salvelinus namaycush). Comparative Biochemistry and Physiology Part C: Toxicology
& Pharmacology.

[22] Zhang S.-Y., Gan X., Shen B., Jiang J., Shen H., Lei Y., Liang Q., Bai C., Huang C., Wu W., Guo Y., Song Y., Chen J. (2023). 6PPD and its metabolite 6PPDQ induce
different developmental toxicities and phenotypes in embryonic zebrafish. Journal of Hazardous Materials.

[23] Hua X., Wang D. (2023). Exposure to 6-PPD Quinone
at Environmentally Relevant Concentrations
Inhibits Both Lifespan and Healthspan in C. elegans. Environ. Sci. Technol..

[24] Kurath G., Garver K. A., Troyer R. M., Emmenegger E. J., Einer-Jensen K., Anderson E. D. (2003). Phylogeography of
infectious haematopoietic
necrosis virus in North America. J. Gen Virol.

[25] Cano-Barbacil C., Radinger J., Argudo M., Rubio-Gracia F., Vila-Gispert A., Garcia-Berthou E. (2020). Key factors explaining critical swimming
speed in freshwater fish: a review and statistical analysis for Iberian
species. Sci. Rep.

[26] Lane R. F., Smalling K. L., Bradley P. M., Greer J. B., Gordon S. E., Hansen J. D., Kolpin D. W., Spanjer A. R., Masoner J. R. (2024). Tire-derived
contaminants 6PPD and 6PPD-Q: analysis, sample handling, and reconnaissance
of United States stream exposures. Chemosphere.

[27] Test No. 203: Fish, Acute Toxicity Test, OECD Guidelines for the Testing of Chemicals; OECD Publishing: Paris, 2019; Section 2.

[28] R Foundation for Statistical Computing; R Core Development Team: Vienna, Austria; 2010.

[29] Ritz C., Baty F., Streibig J. C., Gerhard D. (2015). Dose-Response Analysis Using
R. PLOS ONE.

[30] Kassambara, A. ; Kosinski, M. ; Biecek, P. Survminer: Drawing Survival Curves using ggplot2. R package, version 0.4.9; 2021.

[31] Breyta R., Black A., Kaufman J., Kurath G. (2016). Spatial and temporal
heterogeneity of infectious hematopoietic necrosis virus in Pacific
Northwest salmonids. Infect Genet Evol.

[32] Christensen E. A. F., Stieglitz J. D., Grosell M., Steffensen J. F. (2019). Intra-Specific
Difference in the Effect of Salinity on Physiological Performance
in European Perch (Perca fluviatilis) and Its Ecological Importance
for Fish in Estuaries. Biology (Basel).

[33] Brett J. R. (1964). The respiratory
metabolism and swimming performance of young sockeye salmon. Journal of the Fisheries Board of Canada.

[34] Woytowich, N. ; Roberts, C. ; Ankley, P. ; Brinkmann, M. ; Krogh, E. ; Duncan, K. Uncovering spatially resolved 6PPDQ metabolism in rainbow trout fry with nano-DESI mass spectrometry imaging, Ver 1. ChemRxiv 2025. 10.26434/chemrxiv-2025-k01bd.

[35] Grasse N., Seiwert B., Massei R., Scholz S., Fu Q., Reemtsma T. (2023). Uptake and Biotransformation of the Tire Rubber-derived
Contaminants 6-PPD and 6-PPD Quinone in the Zebrafish Embryo (Danio
rerio). Environ. Sci. Technol..

[36] Liao X.-L., Chen Z.-F., Ou S.-P., Liu Q.-Y., Lin S.-H., Zhou J.-M., Wang Y., Cai Z. (2024). Neurological impairment
is crucial for tire rubber-derived contaminant 6PPDQ-induced acute
toxicity to rainbow trout. Science Bulletin.

[37] Martens K. D., Dunham J. (2021). Evaluating Coexistence of Fish Species with Coastal
Cutthroat Trout in Low Order Streams of Western Oregon and Washington,
USA. Fishes.

[38] Flores A.-M., Davies M. M., Kushneryk K., Lawn P. T. E. S., Helms S., Thomson H. M., Nelson K. R., Burns C. W., Roias S., Gerwing T. G. (2021). Declines of Juvenile Coastal Cutthroat
Trout and Coho Salmon over Fifteen Years in a Salmon-Bearing Stream
in the Salish Sea. Western North American Naturalist.

[39] Hudy, M. ; Roper, B. ; Gillespie, N. Large scale assessments: lessons learned for native trout management. Wild trout IX: sustaining wild trout in a changing world, Wild Trout Symposium, Bozeman, MT, 2007.

[40] Shankar, P. ; Dalsky, E. M. ; Salzer, J. E. ; Greer, J. B. ; Lane, R. F. ; Batts, W. N. ; Gregg, J. ; Kurath, G. ; Hershberger, P. K. ; Hansen, J. D. Evaluation of Lethal and Sublethal Effects of 6PPD-Q on Coastal Cutthroat Trout (Oncorhynchus clarkii clarkii). U.S. Geological Survey Data Release; U.S. Geological Survey, 2024: 10.5066/P16SMKIJ. PMC12177923 40471131

[41] Di S., Liu Z., Zhao H., Li Y., Qi P., Wang Z., Xu H., Jin Y., Wang X. (2022). Chiral perspective evaluations: Enantioselective
hydrolysis of 6PPD and 6PPD-quinone in water and enantioselective
toxicity to Gobiocypris rarus and Oncorhynchus mykiss. Environ. Int..

[42] Dixon P., Paley R., Alegria-Moran R., Oidtmann B. (2016). Epidemiological characteristics
of infectious hematopoietic necrosis virus (IHNV): a review. Veterinary Research.

[43] Parisot T. J., Yasutake W. T., Klontz G. W. (1965). Virus diseases of
the Salmonidae
in western United States. I. Etiology and epizootiology. Ann. N.Y. Acad. Sci..

[44] Lapatra, S. E. ; Williams, S. R. ; Parson, J. E. ; Jones, G. R. ; McRoberts, W. O. Susceptibility of Cutthroat Trout, Rainbow Trout, and Hybrids to Infectious Hematopoietic Necrosis. American Fisheries Society Newsletter; American Fisheries Society, 1994; p 1.

[45] Fang L., Fang C., Di S., Yu Y., Wang C., Wang X., Jin Y. (2023). Oral exposure to tire rubber-derived
contaminant 6PPD and 6PPD-quinone induce hepatotoxicity in mice. Sci. Total Environ..

[46] Fang C., Di S., Yu Y., Qi P., Wang X., Jin Y. (2024). 6PPD induced
cardiac dysfunction in zebrafish associated with mitochondrial damage
and inhibition of autophagy processes. Journal
of Hazardous Materials.

[47] Pearson M., Stevens E. (1991). Size and hematological impact of
the splenic erythrocyte
reservoir in rainbow trout, Oncorhynchus mykiss. Fish physiology and biochemistry.

[48] McIntyre J. K., Prat J., Cameron J., Wetzel J., Mudrock E., Peter K. T., Tian Z., Mackenzie C., Lundin J., Stark J. D., King K., Davis J. W., Kolodziej E. P., Scholz N. L. (2021). Treading Water: Tire Wear Particle
Leachate Recreates an Urban Runoff Mortality Syndrome in Coho but
Not Chum Salmon. Environ. Sci. Technol..

[49] Blair S. I., Barlow C. H., McIntyre J. K. (2021). Acute cerebrovascular effects in
juvenile coho salmon exposed to roadway runoff. Canadian Journal of Fisheries and Aquatic Sciences.

