# Supplementary material for: Evaluation of 6PPD-Quinone Lethal Toxicity and Sublethal Effects on Disease Resistance and Swimming Performance in Coastal Cutthroat Trout (Oncorhynchus clarkii clarkii)
Source: Environ Sci Technol. 2025 Jun 5;59(23):11505–14. doi: 10.1021/acs.est.5c03697 (PMC12177923; doi:10.1021/acs.est.5c03697)
Supplement: Supplementary file 1 [file es5c03697_si_001.pdf]

Supporting Information

Evaluation of 6PPD-Quinone Lethal Toxicity and Sublethal Effects on Disease Resistance and Swimming Performance in Coastal Cutthroat Trout (*Oncorhynchus clarkii clarkii*)

Prarthana Shankar[1*], Ellie M. Dalsky[1], Joanne E. Salzer[1], Rachael F. Lane[2], Sophie Hammond[1], William N. Batts[1], Jacob L. Gregg[3], Justin B. Greer[1], Gael Kurath[1], Paul K. Hershberger[3], and John D. Hansen[1]

1 - U.S. Geological Survey, Western Fisheries Research Center, Seattle WA 98115, USA

2 - U.S. Geological Survey, Kansas Water Science Center, Lawrence KS 66049, USA

3 - U.S. Geological Survey, Western Fisheries Research Center, Marrowstone Marine Field Station, Nordland WA 98358, USA

Summary:

23 pages (Pages S1-S23)

3 supplementary methods (Method SM1-SM3)

7 supplementary tables (Table S1-S7)

11 supplementary figures (Figure S1-S11)

Correspondence to:

Prarthana Shankar, Ph.D.

U.S. Geological Survey, Western Fisheries Research Center, Seattle WA 98115, USA

Email: pshankar@usgs.gov, Telephone: 206-526-6591

Contents

Supplementary Method SM1 – Additional 6PPDQ sensitivity experiments S1
Supplementary Table S1 – Overview of the five sublethal experiments S2
Supplementary Method SM2 – Disease susceptibility methods S3
Supplementary Figure S1 – Swim flume images S5
Supplementary Method SM3 – Swimming performance methods S6
Supplementary Table S2 – Experimental swim performance protocols for Experiments 4 and 5 .... S6
Supplementary Figure S2 – Individual 6PPDQ dose response curves for alevin, fry, parr, and juvenile CCT S8
Supplementary Table S3 – Initial and final analytical measurements for life stage sensitivity determination experiments S9
Supplementary Figure S3 – Loss of 6PPDQ over 24 hours in Experiment 2 .... S10
Supplementary Figure S4 – 24-hour loss of 6PPDQ in tanks without fish S11
Supplementary Figure S5 – Dose response comparison between CCT-Cowlitz and CCT from Eells State hatchery S12
Supplementary Figure S6 – 96-hour loss of 6PPDQ S13
Supplementary Figure S7 – Semi-static 96-hour vs. pulsed exposure to 6PPDQ S14
Supplementary Figure S8 – Disease susceptibility Experiment 1 .... S15
Supplementary Table S4 – Plaque assay for mortalities from Disease susceptibility Experiment 1 .... S16
Supplementary Figure S9 – Disease susceptibility Experiment 2 .... S17
Supplementary Figure S10 – Examples of signs of IHNV infection S19
Supplementary Table S5 – Analytical confirmation of 6PPDQ concentrations in Disease susceptibility Experiments 2 and 3 .... S20
Supplementary Table S6 – LC estimations for alevin, 2-5-week post swim-up fry, 5-month parr, and 13-month juvenile CCT S21
Supplementary Figure S11 – Swim performance step plots S22
Supplementary Table S7 – Analytical confirmation of 6PPDQ concentrations in Swimming performance Experiments 4 and 5 .... S23
Supplementary References S24

Supplementary Method SM1 – Additional 6PPDQ sensitivity experiments

2.4. Additional 6PPDQ-quinone (6PPDQ) sensitivity experiments

Coastal cutthroat trout (CCT; Oncorhynchus clarkii clarkii)-Cowlitz (CCT-Cowlitz) 6PPDQ Sensitivity Determination

CCT-Cowlitz 6PPDQ sensitivity was determined in 8 and 11-month juvenile fish, exposed to nominal concentrations from 100 – 300 ng/L (n = 9 tanks) 10 °C ± 1 °C. At the same time, 20 and 23-month CCT were exposed to 100 – 300 ng/L 6PPDQ (n = 6 tanks). Methods followed the age 13-month juvenile exposures described in the manuscript methods; 6PPDQ concentrations were measured in six of the nine CCT-Cowlitz tanks and three of the six CCT tanks.

Westslope cutthroat trout (WCT; O. clarkii lewisii) 6PPDQ Sensitivity Determination

Methods for 24-hour 6PPDQ exposures to WCT alevin closely followed CCT alevin methods, with the exception that each tank first included 10 alevins (4 concentrations, 500 – 10,000 ng/L), which was followed by one additional 10,000 ng/L exposure to 20 alevins. WCT fry (7-8 weeks post-hatch) were exposed to 500 and 3,000 ng/L 6PPDQ in the same way as the CCT parr. No water samples from the WCT exposures were measured for 6PPDQ concentrations.

CCT Pulsed Exposures

One pulsed exposure study to mimic sequential storms was conducted with age 24-month juvenile CCT at 10 °C ± 1 °C. Fish were exposed to a total of four 24-hour exposures using either 100, 150, or 200 ng/L nominal 6PPDQ pulses over a two-week trial period. Methods for the 24-hour 6PPDQ exposures followed the age 13-month juvenile exposures described in the manuscript methods, and fish were moved to 2-ft circular tanks with flowthrough wet lab water in between exposures. Mortalities were counted and removed daily. 6PPDQ concentrations were not measured in water samples from the pulsed exposure study.

Supplementary Table S1 – Overview of the five sublethal experiments

See excel file, tab 1: "Supplementary tables_6PPDQ CCT.xlsx"

Table S1. Overview of the five sublethal experiments: Experiments 1-3 for disease susceptibility, and Experiments 4 and 5 for measurement of swimming performance. (N/A = not applicable since no 6PPDQ exposure was conducted)

Supplementary Method SM2 – Disease susceptibility methods

2.5. Disease susceptibility test methods (supplement)

Virus exposure and fish sampling

Experiment 1 – Susceptibility of CCT to four different strains of Infectious hematopoietic necrosis virus (IHNV)

Viral subgroups: The Blk94 isolate of IHNV was used to represent the UP subgroup [1], and the DW10, Qts07, and FR0031 isolates were used to represent the UC, MD, and L subgroups, respectively. [2] Viral stocks were amplified in the epithelioma papulosum cyprinid (EPC) cell line using minimal essential media with 10% fecal calf serum, and quantified by plaque assay as previously described. [3]

Virulence: To compare virulence of the four IHNV subgroups in CCT, three tanks of 20 parr fish (1.95 g ± 0.36 standard deviation, SD) were exposed to each of the four viral isolates or a Mock control treatment (virus-free media) by static immersion for 2 hours using a standard high dose of 2 x 10^5 plaque-forming units (PFU)/mL in 1L of wet laboratory water. After immersion, flow-through water (10 °C) was resumed with a total volume of 5L in each tank. Fish were fed three times each week and were monitored daily for 30 days for signs of IHNV infection and mortality. Fish found moribund were euthanized; moribund fish and mortalities were removed daily and stored at -80°C. At the end of the 30-day observation period, all remaining surviving fish were euthanized. All fish that died (n=53) were tested using the plaque assay method for homogenized whole fish tissues [3] to determine the presence and quantity of infectious virus. Goals were to confirm virus as the likely cause of mortality, compare viral loads in the mortalities between the four IHNV subgroups, and determine if any mortalities in the mock groups had detectable virus.

Infection: To compare infectivity of the UP, UC, MD, and L isolates in CCT, an additional tank of 20 fish were challenged with each isolate as described above. For each isolate, 10 fish were sampled on days three and seven post exposure to quantify subclinical infection. The fish were euthanized, and individual head kidney tissue samples were dissected and stored in 500 µL RNALater (Invitrogen, California USA) until processing. RNA extraction and quantification methods generally followed previously published work. [4] Briefly, samples were transferred to Buffer RLT (Qiagen, Hilden, Germany) and homogenized using 1mm zirconia/silica beads (BioSpec Products, Inc., Oklahoma, USA) with a Fast-Prep 24-bead beater (MP Biomedical, California, USA) for 60 seconds. Total RNA was extracted using the RNeasy Mini-kit (Qiagen, Venlo, The Netherlands), following the manufacturer's protocol including the in-column DNase treatment. Total RNA was eluted in 30 µL nuclease-free water and quantified using the Nanodrop ND-1000 (Thermo Fisher Scientific, Massachusetts, USA). Reverse transcription was initiated with 11 µL total RNA using the High-Capacity cDNA kit (Thermo Fisher Scientific, Massachusetts, USA), with added RNase inhibitor (Promega RNasin Plus, Wisconsin, USA). cDNA was diluted 1:5 in nuclease-free water, and RT-qPCR was performed using the IHNV universal N-gene assay [5], which includes an artificial positive control plasmid for generation of a standard curve for quantification of the N-gene. Viral loads are expressed in copies of IHNV RNA/µg kidney RNA.

Experiment 2 - 6PPDQ+IHNV trial 1: Based on results from the 24-hour 6PPDQ parr life stage sensitivity exposures and IHNV Experiment 1, we exposed CCT that weighed 1.95 ± 0.47 g SD to a sublethal concentration of 50 ng/L 6PPDQ for 24 hours, followed by a 24-hour depuration, and then exposed them to the Qts07 isolate (MD subgroup), at the standard high dose used above. Control groups exposed to only chemical or only virus were included, resulting in four treatment groups: Mock, 6PPDQ-only, IHNV-only, and 6PPDQ+IHNV.

6PPDQ exposure: Parr CCT were exposed as four groups of 40 fish each in the 20-gallon glass aquaria to 50 ng/L 6PPDQ (nominal). Similar to our previous methods for 6PPDQ exposures, fish were exposed in a volume of 65L. Four additional control tanks with 40 fish/tank were also included and exposed to DMSO only, at the DMSO % in the 6PPDQ exposure tanks. Aquaria were covered with mesh nets, and fish were left undisturbed for 24 hours. After chemical exposure, any

fish showing acute toxicity effects were removed and euthanized. The remaining fish were moved to tanks with flow-through wet laboratory water for 24 hours.

Virulence: To investigate differences in disease susceptibility of CCT to IHNV after exposure to 6PPDQ, the chemical-exposed fish were challenged with the Qts07 isolate as described in Experiment 1 (n = 19 fish/tank); fish were monitored daily for 35 days for signs of disease and mortality, and all moribund fish and mortalities were collected daily and stored at -80°C. Forty-six of the 109 mortalities were tested by plaque assay as described above; **data are not shown** since no differences were observed between the two IHNV groups, and the Mock and 6PPDQ-only groups were virus free.

Infection: To investigate IHNV infection differences between the IHNV and 6PPDQ-IHNV groups, an additional replicate tank of 19-20 fish for each treatment combination was set up, and 9-10 fish were sampled on days 1 and 3 post viral exposure. Similar to Experiment 1, individual head kidney samples were processed for IHNV viral load quantification.

Experiment 3 - 6PPDQ+IHNV trial 2:

6PPDQ exposure: 6PPDQ exposure with larger parr fish (3.72 ± 0.90 g SD) closely followed methods described in Experiment 2 but using 75ng/L nominal concentration of 6PPDQ for 24 hours, with fish directly transferred to larger virus challenge tanks without the 24-hour depuration period that was used in Experiment 2. Four control tanks (n=40 fish/tank) were included to test exposure to only DMSO as described above.

Virulence: In Experiment 3, DMSO- and 6PPDQ-exposed fish (n = 20-21 fish/tank) were challenged in larger tanks by immersion in 2 x 10^5 PFU/mL of Qts07 virus isolate in 5L of wet laboratory water for 2 hours. After immersion, flow-through wet laboratory water (10 °C) was resumed with a total volume of 24L in each tank. Fish were observed daily for 36 days for signs of disease and mortality. Similar to Experiments 1 and 2, all moribund fish and mortalities were collected daily and stored at -80°C; in Experiment 3, 15 of the 22 mortalities were tested by plaque assay and we confirmed IHNV as the likely cause of mortalities in the two IHNV treatment groups (IHNV and 6PPDQ+IHNV) (**data not shown**).

Infection: Methods closely followed those described in Experiment 2 with 10 fish kidneys per treatment sampled each on days 1 and 3 post IHNV challenge.

Of note, we observed higher levels of mortality than expected in the Mock treatment fish in both Experiments 1 and 2, with 88.3 % or 80.7% survival at the end of the observation periods, respectively (**Supplementary Figures S8A and S9A**). Moribund and dead fish from mock and virus-exposed groups in both experiments included many with clinical signs that were not consistent with IHN infection, but instead ranged from minor erosion to complete absence of the caudal fins. These signs are typical of Flavobacterial infections that are common in salmonid fish but become opportunistic and act in synergy with IHNV under stressful conditions. [6, 7] This had greatest impact in Experiment 2, where “tail-rot” clearly contributed to the unexpectedly low survival in all groups. We reasoned that cutthroat trout are less domesticated than rainbow trout (*Onchorhynchus mykiss*) and may be less tolerant of confinement in our standard 5-L challenge tanks, leading to increased stress as has been previously suggested. [8] Therefore, in Experiment 3, we increased volume of water used for the two-hour viral immersion to 5 L (1 L in Experiments 1 and 2) and held fish in a total volume of 24 L (5 L in Experiments 1 and 2) for the observation period. These changes reduced fish densities and likely contributed to the higher survival of all fish in Experiment 3, with 100% survival of the Mock treatment fish (**Figure 3A**).

Supplementary Figure S1 – Swim flume images

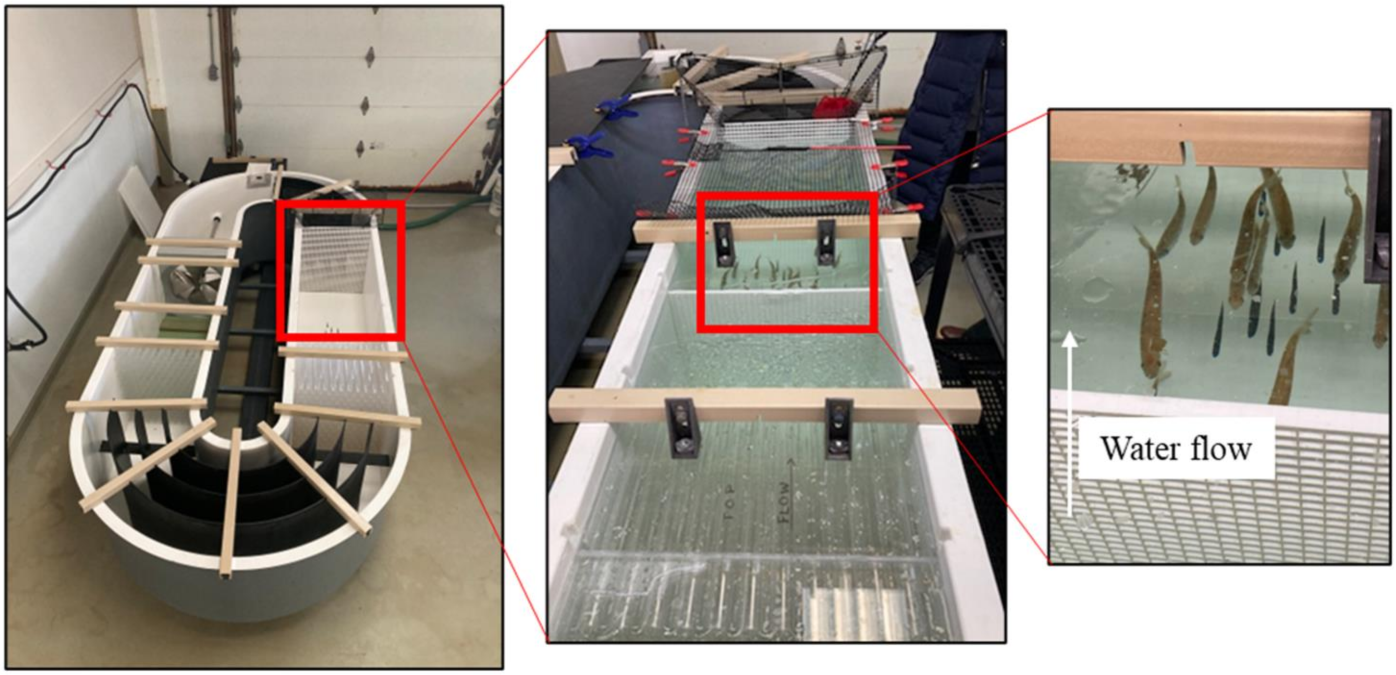

Figure S1. Example images of custom-built swim flume at the U.S. Geological Survey, Marrowstone Marine Field Station

Supplementary Method SM3 – Swimming performance methods

2.6. Swimming performance test methods

Determination of swim performance protocols

Two to four pilot studies were conducted before each experiment (Experiment 4 with ~15-month-old CCT, Experiment 5 with ~24-month-old CCT) to determine the ramping parameters for the fish of the two sizes. All body lengths (BL) listed below were first approximately calculated for the pilot trials, and then converted to reflect measured BLs of the fish in the experimental trials. Pilot trials were conducted with the goal of finding at least an hour-long protocol when fish fatigued over at least two velocity increment steps allowing reasonable time between individual fish fatiguing to not overwhelm the experimenter collecting the fatigued fish.

The four pilot trials for Experiment 4 (CCT had an approximate fork length = 105 mm), with a summary of the results, were as follows:

1. Water velocity increments of 1 BL/sec from 0 to 6 every 15 mins: *Overall protocol too long; fish did not fatigue until two hours into the assay, and fish did not orient against the water flow at 1 BL/sec.*
2. Water velocity increments of 1 BL/sec from 2 to 4 every 5 mins, followed by 5 to 7 every 15 mins: *Fish fatiguing happened too quickly; 55% of the fish fatigued at 5 BL/sec.*
3. Water velocity increments of 1 BL/sec from 2 to 4 every 10 mins, followed by 5 to 7 every 15 mins: *Fish began to fatigue at 5 BL/sec but 85% of the fish fatigued at 6 BL/sec.*
4. Water velocity increments of 1 BL/sec from 2 to 4 every 10 mins, followed by increments of 0.5 BL/sec from 4 to 6 every 15 mins*: Final swim protocol with fish fatiguing across 4.5, 5, and 5.5 BL/s is seen in **Supplementary Table S2.***

The two pilot trials for Experiment 5 (CCT had an approximate fork length = 135 mm), with a summary of the results, were as follows:

1. Water velocity increments of 1 BL/sec from 2 to 4 every 10 mins, followed by increments of 0.5 BL/sec from 4 to 6 every 15 mins: Overall protocol too fast; all 16 tested *fish fatigued in under 40 minutes.*
2. Water velocity increments of 1 BL/sec from 1.5 to 3.5 every 10 mins, followed by increments of 0.5 BL/sec from 3.5 to 5.5 every 15 mins: *Final swim protocol with fish fatiguing across 4 and 4.5 BL/s is seen in **Supplementary Table S2.***

Supplementary Table S2 – Experimental swim performance protocols for Experiments 4 and 5

See excel file, tab 2: "Supplementary tables_6PPDQ CCT.xlsx"

Table S2. Water velocity ramping parameters that were utilized for 6PPDQ-exposed coastal cutthroat trout (CCT; *Oncorhynchus clarkii clarkii*). Total protocol time was 1 hour and 15 min for each test. CCT in Experiment 4 had fork length of 115.82 ± 8.01 mm standard deviation (SD) and in Experiment 5 had fork length of 147.17 ± 15.0 mm SD.

Fish tagging

Approximately two weeks prior to experimental fish transport from the U.S. Geological Survey Western Fisheries Research Center in Seattle to the Marrowstone Field Station, CCT were tagged with either yellow or red visible implant elastomer tags (Northwest Marine Technology, Inc) following manufacturer's protocols. Briefly, fish were anesthetized and placed on a flat surface for tagging. The needle with the elastomer tag was inserted laterally in the operculum and gradually withdrawn as the injector plunger was depressed to eject the elastomer tag. Any excess elastomer was gently wiped off the fish, and fish were immediately returned to fresh water for recovery. All fish in this study were tagged on the left side for consistency.

Fish transport and timeline

The tagged fish for each test were transported to the Marrowstone Field Station in wet laboratory water aerated with oxygen. Upon arrival, fish were moved to 2ft tanks for 1-2 nights of acclimation in Marrowstone lab freshwater at approximately 10 °C ± 1 °C. On the day after fish transport, a subset of the fish was used for practice swim trials for determination of the swim test protocol (described above). The remaining fish were used for a total of six experimental swim trials. Half the fish were set up for a 22-hour 6PPDQ exposure (n=3 trials with eight controls and eight treated fish each) on day 1 followed by swimming performance tests on day 2 after fish transport. The other half were exposed to 6PPDQ for 22 hours starting on day 2 after fish transport followed by swimming performance tests (n=3 trials with eight controls and eight treated fish each) on day 3. On each day, 6PPDQ exposures began at different times such that the 22-hour-post-exposure swim performance trials were all conducted between 9:00 am and 3:00 pm the following day.

6PPDQ chemical exposure

CCT with red elastomer tags were exposed to a nominal concentration of 100 ng/L (Experiment 4; CCT were ~15 months, 14.9 g ± 2.79 g SD, with 115.82 ± 8.01 mm SD fork length) or 150 ng/L (Experiment 5; CCT were ~24 months, 29.4 ± 8.73 g SD with 147.17 ± 15.0 mm SD fork length) 6PPDQ in a static system for 22 hours similar to the methods described in "13-month juvenile exposures" in the manuscript. Control fish (yellow tags) were handled in a similar manner as the 6PPDQ-exposed fish and were exposed to the same DMSO concentration as the DMSO in the 6PPDQ exposure tanks (solvent control). Chemical exposures in 65L volume with 8 fish/treatment (Experiment 4: 1.83 g/L fish loading, Experiment 5: 3.62 g/L fish loading) were conducted on two consecutive days (days 1 and 2 after fish transport), and fish were fasted for ~72 hours or ~96 hours, respectively, prior to exposure. Aquaria were covered with netting and aerated with a single air stone per aquarium. Three each of solvent control and 6PPDQ exposure tanks were set up at different times on day 1, and three pairs of tanks were set up on day 2.

Experimental swim trials

Swimming performance tests were conducted in a custom-built swim flume (**Supplementary Figure S1**). The flume has a propellor on one end whose speed can be adjusted to increase or decrease water flow rates allowing measurement of swimming performance of multiple fish at once. A mixed group of control and 6PPDQ-exposed fish, were placed in the swim flume, and water velocity was gradually increased over the duration of up to 1 hour 15 mins in a manner similar to the swimming respirometry trial method described previously. [9] A fish was considered as fatigued if it was no longer able to orient itself against the flow of water and was repeatedly pushed against the back screen of the swim chamber.

Fish performance was tested 22 hours after the start of chemical exposure. Eight control fish and eight 6PPDQ-exposed CCT (n = 3 trials/day) were rinsed with fresh water, immediately introduced into the swim flume chamber, and the final swim protocol (**Table 1**) began. As individuals fatigued, fish were removed from the swim chamber and the time of "failure" was noted. Fish were euthanized individually, and after the conclusion of the swim performance test, the color of the elastomer tag was noted, and the fork length (cm) and mass (g) were measured. Water temperature, salinity, and dissolved oxygen were recorded in the swim flume at the start and end of each trial, and they were consistently 10 °C ± 1 °C, 0.3 ppt, and 11.0 mg/L ± 0.2 mg/L SD (99.0 % ± 1.0 % SD), respectively, for all trials.

Supplementary Figure S2 – Individual 6PPDQ dose response curves for alevin, fry, parr, and juvenile CCT

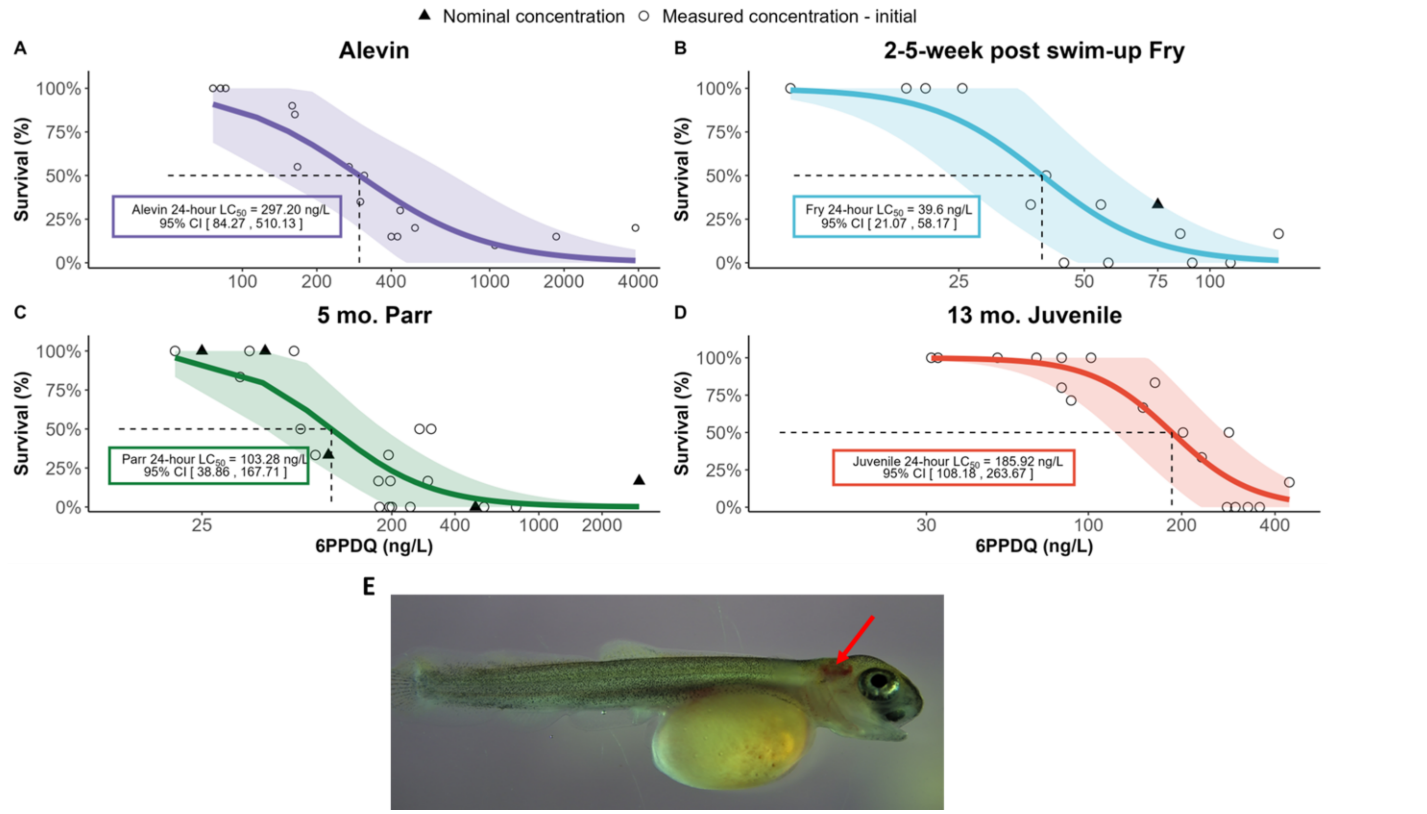

Figure S2. Individual 24-hour (6PPD-quinone) 6PPDQ survival dose response curves for coastal cutthroat trout (CCT; *Oncorhynchus clarkii clarkii*) alevin (A), 2-5-week post swim-up fry (B), 5-month parr (C), and 13-month juvenile fish (D). Figures are data presented in Figure 1B of the manuscript, with the addition of 95% confidence intervals represented by the shaded area around each curve. **(E)** Example image of a CCT alevin showing internal blood hemorrhaging in the brain.

Supplementary Table S3 – Initial and final analytical measurements for life stage sensitivity determination experiments

See excel file, tab 3: “Supplementary tables_6PPDQ CCT.xlsx”

Table S3. Table shows the initial (0 hours) and final (24 hours) nominal and measured concentrations of the individual tanks for the 24-hour life stage sensitivity determination alevin, 2-5-week post swim-up fry, 5-monthparr, and 13-month juvenile exposures. (* and samples in red = final concentrations > initial concentrations, so nominal concentration used for dose response analysis)

Supplementary Figure S3 – Loss of 6PPDQ over 24 hours in Experiment 2

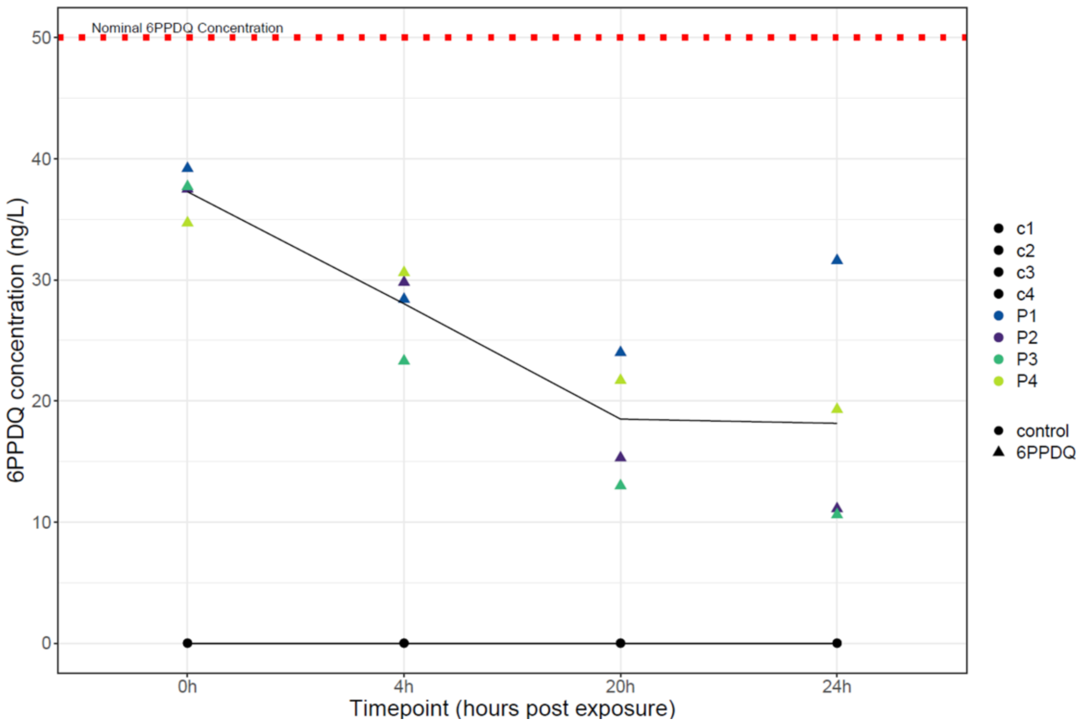

Figure S3. Disease Susceptibility Experiment 2 analytical chemistry confirmation of 6PPD-quinone (6PPDQ) concentrations at 0, 4, 20, and 24h after start of exposure. Samples were collected from four replicate tanks at each time point. c1-c4 are the control tanks (with vehicle control, DMSO), and P1-P4 are the four 6PPDQ tanks with nominal concentration of 50 ng/L. Each tank had a total volume of 65 L with an average fish loading of 1.2 g/L per tank.

Supplementary Figure S4 – 24-hour loss of 6PPDQ in tanks without fish

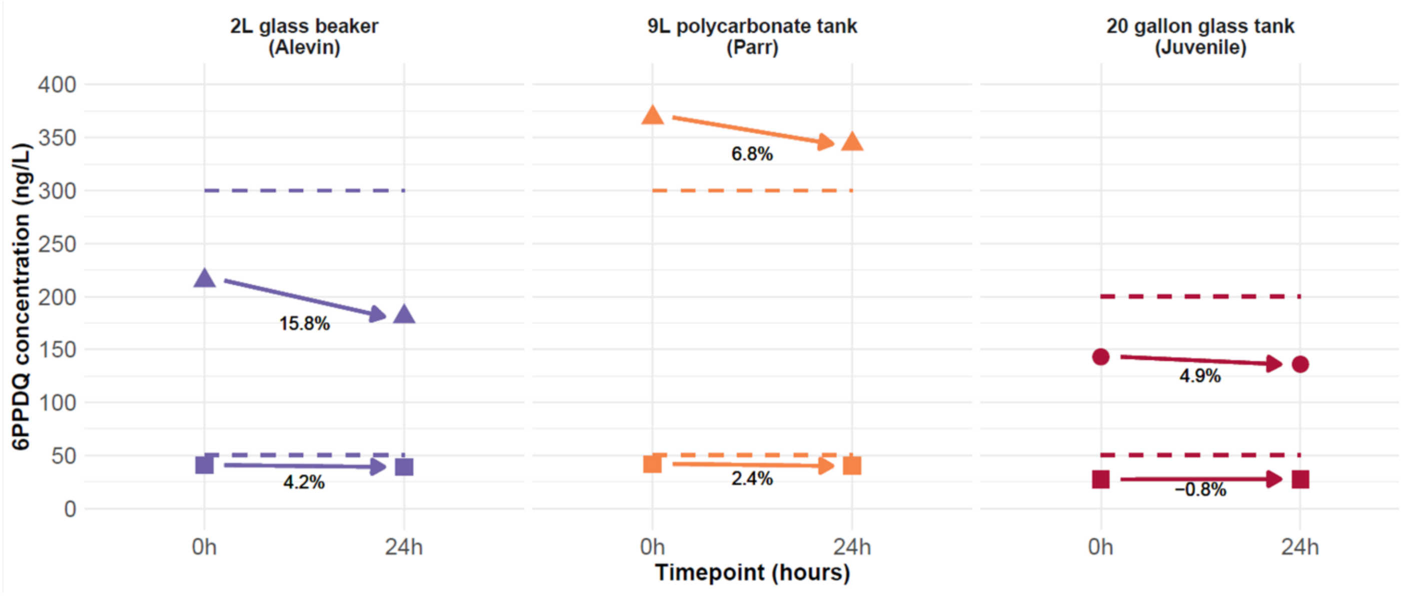

Figure S4. Percent loss of measured 6PPD-quinone (6PPDQ) over 24 hours without fish in the three types of tanks used for the life stage sensitivity determination experiments. The tanks used were 2 L beakers for the alevin exposures, 9 L polycarbonate tanks for the 5-month parr exposures, and 20-gallon glass aquaria for the 13-month juvenile exposures. Dashed lines represent the nominal concentrations that were tested; 50 and 300 ng/L in the 2 L beakers and 9 L tanks, and 50 and 200 ng/L in the 20-gallon aquaria. Percent loss of 6PPDQ over the 24 hours was always under 10%, with the exception of the 300 ng/L nominal 6PPDQ exposure in the 2L glass beaker.

Supplementary Figure S5 – Dose response comparison between CCT-Cowlitz and CCT from Eells State hatchery

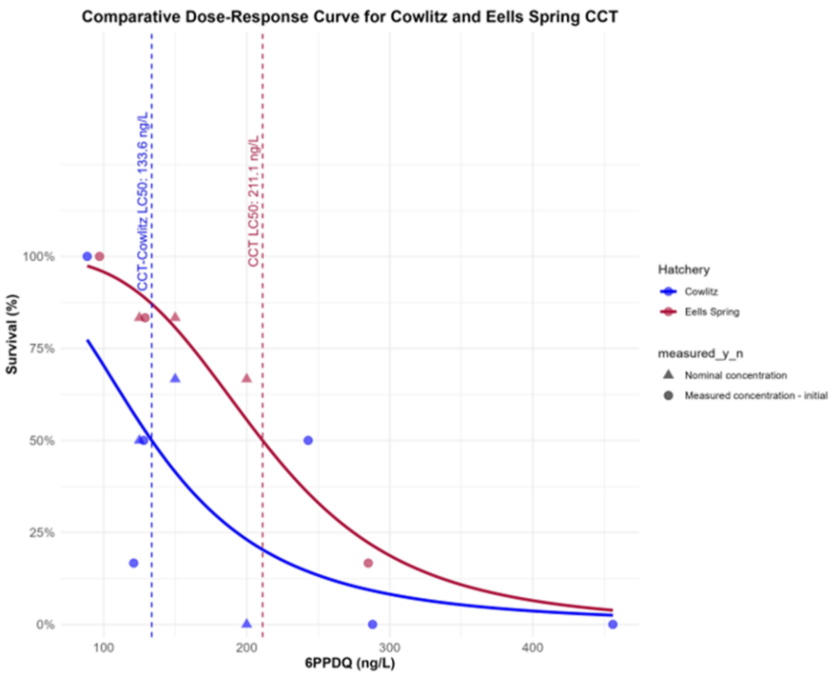

Figure S5. Dose-response curve comparing percent survival of juvenile coastal cutthroat trout (CCT; *Oncorhynchus clarkii clarkii*) and CCT-Cowlitz. 6PPD-quinone (6PPDQ) concentrations are presented on the X-axis, and estimated 24-hour median lethal concentration (LC_{50}) values for each CCT population are depicted by the vertical dotted lines. Points are the percent survival from each 6PPDQ treatment tank (CCT: 20-23-month; n = 6 replicates, CCT-Cowlitz: age 8-11-month; 15.56 g ± 2.45 g, fork length 11.37 cm ± 0.92 cm; n = 9 replicates); circles are initial measured concentrations, and triangles are the nominal concentrations when the measured concentrations were not available.

Supplementary Figure S6 – 96-hour loss of 6PPDQ

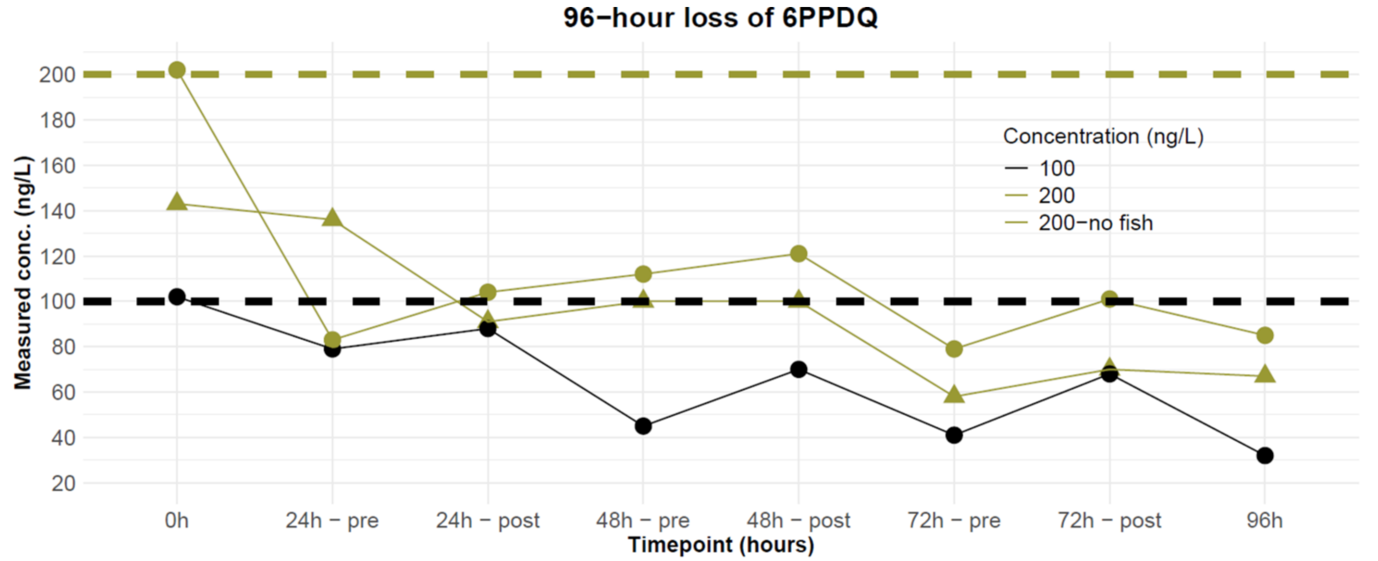

Figure S6. Loss of 6PPD-quinone (6PPDQ) measured in juvenile exposure tanks with (100 and 200 ng/L nominal; dashed lines) and without (200 ng/L nominal) fish over 96 hours. 50% of the exposure solution was renewed every day (24, 48, and 72 hours), and 6PPDQ was analytically measured at 0h, 96h, and pre- and post-renewal at 24, 48, and 72 hours. Triangles represent the 200 ng/L tank without fish. Circles represent tanks with fish.

Supplementary Figure S7 – Semi-static 96-hour vs. pulsed exposure to 6PPDQ

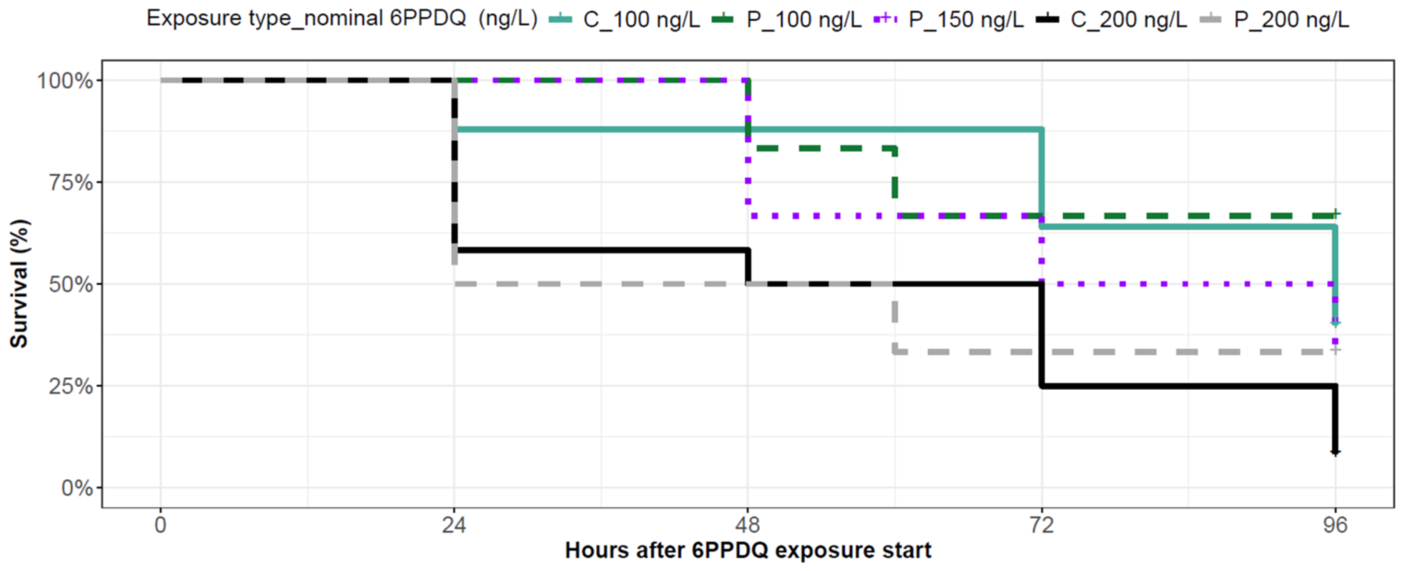

Figure S7. Comparison between semi-static 96-hour 6PPD-quinone (6PPDQ) exposures and pulsed exposures in juvenile coastal cutthroat trout (CCT; *Oncorhynchus clarkii clarkii*). CCT (n=6/tank) were exposed to a total of four 24-hour exposures using either 100, 150, or 200 ng/L nominal 6PPDQ pulses. Exposures were twice per week for two weeks (four total exposures) with freshwater flow-through between exposures. Mortalities during the two-week period were removed daily. Figure compares the percent survival of the pulsed exposures (dashed lines, "P" in legend) to the average percent survival of 13-month juvenile CCT from the 96-hour semi-static exposures shown in Figure 2A, but with using nominal 6PPDQ concentrations (continuous line, "C" in legend). We did not conduct semi-static 96-hour exposures to 150 ng/L 6PPDQ.

Supplementary Figure S8 – Disease susceptibility Experiment 1

In Experiment 1, immersion exposures to a standard high dose of each of the viruses caused low to moderate levels of mortality (**Supplementary Figure S8A**). CCT exposed to the MD isolate, Qts07, showed the lowest average survival of 71.7% across the three replicates (**Supplementary Figure S8A**). Exposure to the L isolate led to 76.7% average survival, followed by 80% with UP, and 95% with UC. The virus was recovered using a plaque assay from 16/17 fish that died after exposure to MD, with 15 (93.75%) of the virus levels considered high titer (> 10^5 PFU/g) (**Supplementary Table S4**). In contrast, only 62.5%, 0%, and 77.8% of the virus-positive mortalities from the UP, UC, and L viral challenges had high titer viral loads.

In addition to mortality and viral loads in mortalities we observed clinical signs consistent with IHN disease. For example, among the MD-infected mortalities 5/17 showed hemorrhaging and 1/17 showed skin darkening prior to mortality (**Supplementary Figure S10** with example images; **Supplementary Table S4**). Similar frequencies of these signs were observed among the UP and L-exposed fish. The mock and UC-exposed groups had smaller numbers of mortalities and no clinical signs were observed.

To evaluate differences in the infectivity and early clearance of the four IHNV strains in CCT, fish head kidneys were sampled on days 3 and 7 to quantify viral loads using RT-qPCR. MD-challenged fish were 100% positive on both days 3 and 7 (n=10/day), and the mean viral load was 4.85 $\log_{10}$ copies/µg RNA on day 3, decreasing to 2.89 $\log_{10}$ copies/µg RNA on day 7 (**Supplementary Figure S8B**). UP and UC challenge fish showed similar high frequencies of virus-positive fish on day 3 but with lower viral loads, and they both showed evidence of clearance on day 7. In contrast, the L challenge fish had much lower frequencies of 3/10 and 4/10 virus-positive fish on days 3 and 7, with viral loads lower than those of the MD group fish.

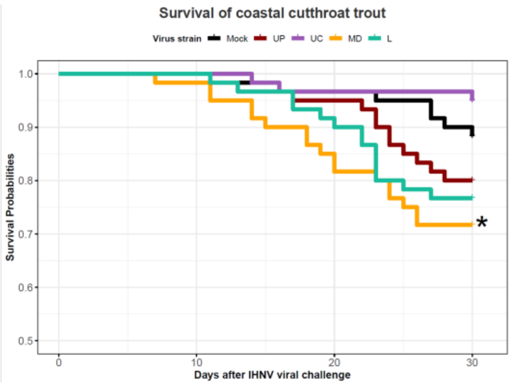

Figure S8. Comparison of coastal cutthroat trout (CCT; *Oncorhynchus clarkii clarkii*) susceptibility to four strains of Infectious hematopoietic necrosis virus (IHNV) (**A**) Daily cumulative percent survival of 5-month parr CCT exposed to UP, UC, MD, and L strains of IHNV or virus-free media (Mock). Fish were exposed to a standard high dose of 2 x 10^5 plaque forming units(PFU)/mL of each strain in a 2-hour static immersion challenge. Survival curves were constructed by pooling data from triplicate groups (n=19) of each treatment. Asterisk indicates that the MD strain had significantly lower survival compared to fish in the Mock treatment ($p < 0.05$, Cox proportional hazard analysis). No significant differences were observed between the other strains (UP, UC, and L) compared to the Mock control group.

(B) Summary of CCT infection frequencies and $\log_{10}$ viral loads in 10 fish per IHNV subgroup (Mock, UP, UC, MD, and L) on days 3 and 7 post viral challenge. Different letters in parentheses indicate treatment groups with mean log viral loads that are significantly different from each other on each day, and asterisk indicates statistical significance between days 3 and 7 of the same strain ($p < 0.05$; Two-way ANOVA; Tukey posthoc test).

**IHNV genogroup**	**IHNV strain**	**Day 3**			**Day 7**		
		Virus positive fish/total fish	Mean log viral load (copies/µg RNA)	Standard deviation	Virus positive fish/total fish	Mean log viral load (copies/µg RNA)	Standard deviation
Mock	N/A	0/10	0 (a)	0	0/10	0 (a)	0
UP	Blk94	9/10	2.57 (b)	1.14	6/10	0.88 (a)	0.30
UC	DW10	8/10	2.15 (b)	1.57	4/10	0.97 (a)	0.82
MD	Qts07	10/10	4.85 (c)	1.02	10/10	2.89 (b,*)	1.70
L	FR0031	3/10	2.66 (b)	1.00	4/10	1.87 (a,b)	1.56

Supplementary Table S4 – Plaque assay for mortalities from Disease susceptibility Experiment 1

See excel file, tab 4: “Supplementary tables_6PPDQ CCT.xlsx”

Table S4. Plaque assay results to determine the presence and quantity of each Infectious hematopoietic necrosis virus (IHNV) genogroups in Experiment 1 mortalities.

Supplementary Figure S9 – Disease susceptibility Experiment 2

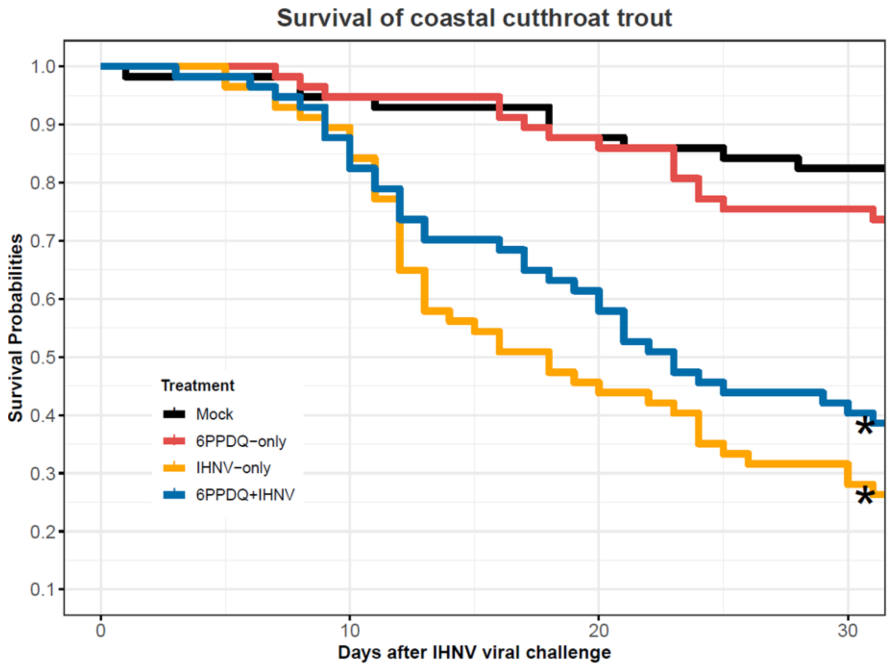

Figure S9. (A) Daily cumulative percent survival of 7-month coastal cutthroat trout (CCT; *Oncorhynchus clarkii clarkii*) following exposure to 6PPD-only, IHNV-only, 6PPDQ+IHNV, and a Mock treatment group in Experiment 2. The 6PPDQ exposure was to 50 ng/L (nominal) for 24 hours. After a 24-hour depuration period, the virus exposure followed, and was a two-hour static immersion challenge to 2 x 10^5 plaque forming units (PFU)/mL of the MD genogroup (Qts07 isolate). The Mock group (control) was exposed to equivalent % DMSO as the 6PPDQ treatment, followed by virus-free media during the viral challenge. Survival curves were constructed by pooling data from triplicate groups (n=19) of each treatment. Asterisk indicates that the two IHNV groups had significantly lower survival compared to fish in the mock treatment ($p < 0.05$, Cox proportional hazard analysis). No significant differences were observed between the two IHNV groups (IHNV-only and 6PPDQ+IHNV), or between 6PPDQ-only and the Mock groups.

(B) Summary of CCT infection frequencies and $\log_{10}$ viral loads in 9-10 fish/treatment group (Mock, 6PPDQ-only, IHNV-only, 6PPDQ+IHNV) on days 1 and 3 post viral challenge. Different letters in parentheses indicate treatment groups with mean log viral loads that are significantly different from each other on each day, and asterisk indicates statistical significance between days 1 and 3 of the same treatment group ($p < 0.05$; Two-way ANOVA; Tukey posthoc test).

Treatment	Day 1			Day 3		
	Virus positive fish/total fish	Mean log viral load (copies/µg RNA)	Standard deviation	Virus positive fish/total fish	Mean log viral load (copies/µg RNA)	Standard deviation
Mock	1/10	3.09 (a)	N/A	5/10	1.12 (a)	0.78
6PPDQ	0/9	0 (a)	0	0/10	0 (a)	0
IHNV	9/10	2.10 (b)	0.80	10/10	5.65 (b,*)	1.75
6PPDQ+IHNV	8/9	1.74 (b)	0.85	9/9	5.84 (b,*)	1.74

Supplementary Figure S10 – Examples of signs of IHNV infection

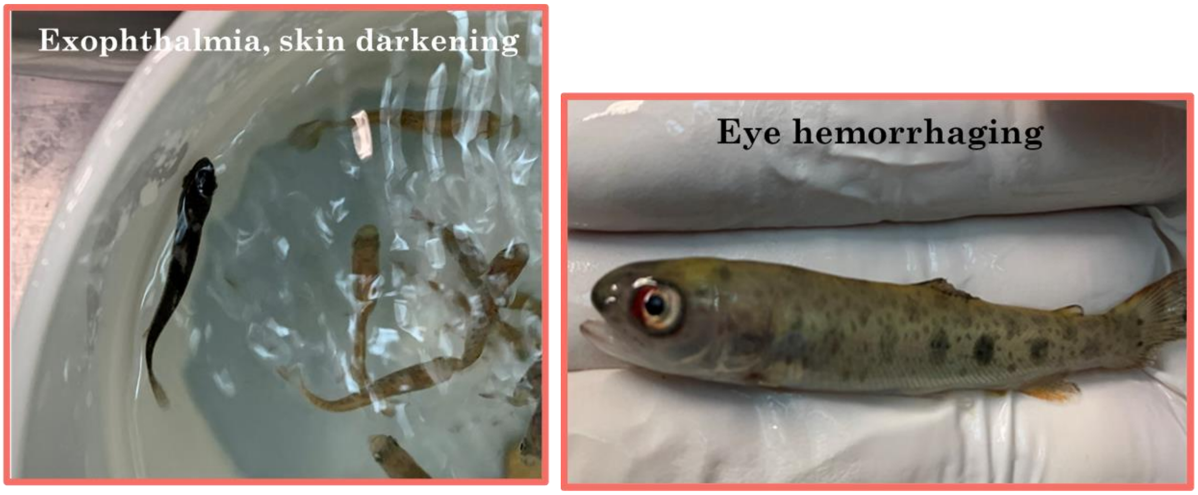

Figure S10. Examples of signs of viral infection observed in the Infectious hematopoietic necrosis virus (IHNV) (MD-strain)-infected juvenile coastal cutthroat trout in Experiments 1, 2, and 3.

Supplementary Table S5 – Analytical confirmation of 6PPDQ concentrations in Disease susceptibility Experiments 2 and 3

See excel file, tab 5: “Supplementary tables_6PPDQ CCT.xlsx”

Table S5. Analytical verification of 6PPD-quinone (6PPDQ) in 24-hour exposures (only initial measurements) conducted for the Disease Susceptibility Experiments 2 and 3. (n.m. = not measured, N/A = not applicable)

Supplementary Table S6 – LC estimations for alevin, 2-5-week post swim-up fry, 5-month parr, and 13-month juvenile CCT

See excel file, tab 6: “cutthroat trout_6ppdq_supplementary tables_2025MAR19.xlsx”

Table S6. Lethal concentration (LC) estimations for alevin, 2-5-week post swim-up fry, 5-month parr, and 13-month juvenile coastal cutthroat trout (CCT; *Oncorhynchus clarkii clarkii*) based on Figure 1A.

Supplementary Figure S11 – Swim performance step plots

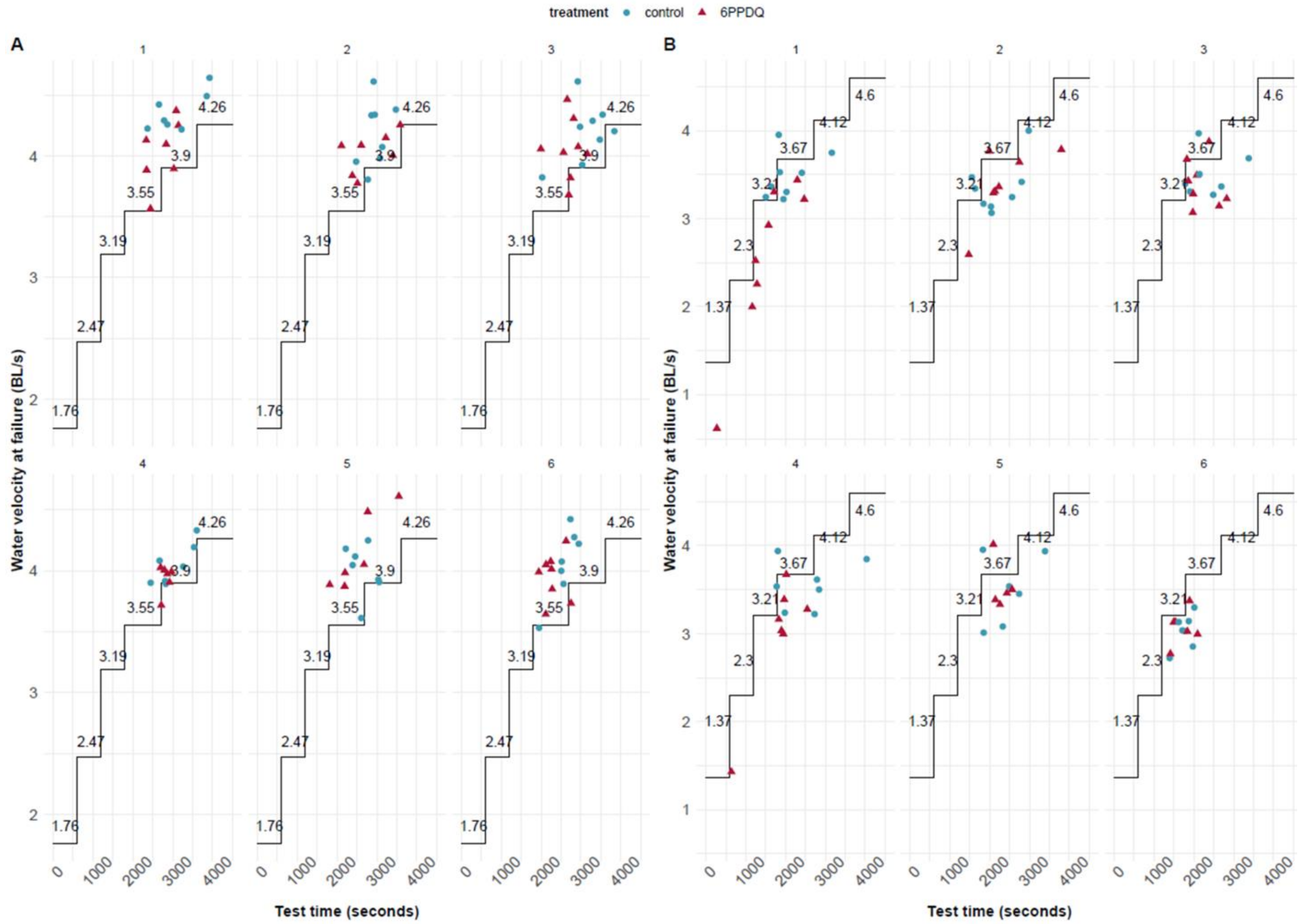

Figure S11. Experiment 4 (72.2 ng/L 6PPD-quinone [6PPDQ]; 15-month coastal cutthroat trout [CCT; *Oncorhynchus clarkii clarkii*]) and (C) Experiment 5 (120.5 ng/L 6PPDQ; 24-month CCT) step-plots showing water velocity (BL/S) relative to test time (seconds) overlayed with the individual fish fail times that were standardized by individual body length. Data for each of the six trials (number at the top of each graph) are presented.

Supplementary Table S7 – Analytical confirmation of 6PPDQ concentrations in Swimming performance Experiments 4 and 5

See excel file, tab 7: "Supplementary tables_6PPDQ CCT.xlsx"

Table S7. Analytical verification of 6PPD-quinone (6PPDQ) in 22-hour exposures conducted before testing swimming performance of 18-month or 24-month juvenile coastal cutthroat trout (CCT; *Oncorhynchus clarkii clarkii*). (n.m. = not measured, N/A = not applicable).

Supplementary References

1. Garver, K.A., Batts, W.N., and Kurath, G., Virulence Comparisons of Infectious Hematopoietic Necrosis Virus U and M Genogroups in Sockeye Salmon and Rainbow Trout. *Journal of Aquatic Animal Health*, **2006**. 18(4): p. 232-243. DOI: 10.1577/H05-038.1
2. Paez, D.J., McKenney, D., Purcell, M.K., Naish, K.A., and Kurath, G., Variation in within-host replication kinetics among virus genotypes provides evidence of specialist and generalist infection strategies across three salmonid host species. *Virus Evol*, **2022**. 8(2): p. veac079. DOI: 10.1093/ve/veac079
3. Batts, W. and Winton, J., Enhanced detection of infectious hematopoietic necrosis virus and other fish viruses by pretreatment of cell monolayers with polyethylene glycol. *Journal of Aquatic Animal Health*, **1989**. 1(4): p. 284-290. DOI: 10.1577/1548-8667(1989)001<0284:EDOIHN>2.3.CO;2
4. Paez, D.J., Kurath, G., Powers, R.L., Naish, K.A., and Purcell, M.K., Local and systemic replicative fitness for viruses in specialist, generalist, and non-specialist interactions with salmonid hosts. *J Gen Virol*, **2024**. 105(1). DOI: 10.1099/jgv.0.001937
5. Purcell, M.K., Thompson, R.L., Garver, K.A., Hawley, L.M., Batts, W.N., Sprague, L., Sampson, C., and Winton, J.R., Universal reverse-transcriptase real-time PCR for infectious hematopoietic necrosis virus (IHNV). *Diseases of Aquatic Organisms*, **2013**. 106(2): p. 103-115. DOI: 10.3354/dao02644
6. Ma, J., Bruce, T.J., Oliver, L.P., and Cain, K.D., Co-infection of rainbow trout (Oncorhynchus mykiss) with infectious hematopoietic necrosis virus and Flavobacterium psychrophilum. *J Fish Dis*, **2019**. 42(7): p. 1065-1076. DOI: 10.1111/jfd.13012
7. Starliper, C.E., Bacterial coldwater disease of fishes caused by Flavobacterium psychrophilum. *Journal of Advanced Research*, **2011**. 2(2): p. 97-108. DOI: 10.1016/j.jare.2010.04.001
8. Klug, J.J., Treuting, P.M., Sanders, G.E., Winton, J.R., and Kurath, G., Effects of Stocking Density on Stress Response and Susceptibility to Infectious Hematopoietic Necrosis Virus in Rainbow Trout. *J Am Assoc Lab Anim Sci*, **2021**. 60(6): p. 637-645. DOI: 10.30802/aalas-jaalas-21-000003
9. Christensen, E.A.F., Stieglitz, J.D., Grosell, M., and Steffensen, J.F., Intra-Specific Difference in the Effect of Salinity on Physiological Performance in European Perch (Perca fluviatilis) and Its Ecological Importance for Fish in Estuaries. *Biology (Basel)*, **2019**. 8(4): p. 89. DOI: 10.3390/biology8040089
